# Phenological modeling for ecological double-cropping of grapes with rain and pest resilience strategies based on MobileNet developed by Augmented Dream Optimizer

**DOI:** 10.3389/fpls.2026.1779481

**Published:** 2026-04-13

**Authors:** Xiaojing Gan

**Affiliations:** Faculty of Pharmacy, Guangxi University of Chinese Medicine, Nanning, China

**Keywords:** Augmented Dream Optimizer, CropHarvest, deep learning, double-cropping viticulture, MobileNet, phenological modeling, precision agriculture

## Abstract

Double-cropping grape systems offer enhanced land productivity but face significant challenges from climate variability, particularly rain stress and pest outbreaks during critical phenological stages. Accurate phenological prediction is essential to synchronize management practices with crop development and improve ecological resilience. This study presents a novel deep learning framework that integrates MobileNet with an augmented version of Dream Optimizer [Augmented Dream Optimizer (ADO)] to model grape phenology using satellite-derived time series of Normalized Difference Vegetation Index (NDVI), Enhanced Vegetation Index (EVI), and rainfall from the CropHarvest dataset. The model transforms temporal data into pseudo-images for efficient spatiotemporal feature extraction, achieving 93% classification accuracy and a 6.1-day mean absolute error in stage prediction. ADO enhances convergence and generalization by optimizing key hyperparameters through a hybrid metaheuristic search. The system further identifies high-risk periods for rain damage and pest infestation, enabling proactive interventions. The model statistically significantly outperforms machine learning and deep learning baselines (p< 0.01) across three different agroecological zones [Mediterranean (California, USA, and southern Europe), subtropical (South Australia), and temperate (central Europe)] through spatially stratified fivefold cross-validation on 3,000 held-out test samples of the CropHarvest dataset. This work demonstrates the potential of optimized lightweight neural networks in sustainable viticulture, providing a scalable tool for precision management in climate-resilient double-cropping systems.

## 1 Introduction

Grape growing is a significant agricultural industry worldwide, representing both the foundation of the global wine industry and the markets for fresh fruit (Fatchiyah et al., 2025). In recent years, the double-cropping system has received growing interest, as it can potentially optimize land use, maximize total yield per unit area, and shorten growing cycles. Under double-cropping, a commercially viable crop is grown in succession or in parallel on the same land at some point in a year (Piña-Rey et al., 2021).

This approach is particularly attractive for the regionally constrained and economically sensitive grape-growing regions because producers could likely optimize their production cycles to obtain a greater return on their investment (Rafique et al., 2024). The application of double-cropping systems in vineyards creates a few complications and challenges with regard to managing crop growth processes and environmental interactions (Bani et al., 2025).

One of the main limitations in viticulture is stress from fluctuating rainfall associated with pest attacks, which can be chronic under many of the same environmental conditions exacerbated by climate variability. Not enough rain, too much rain, and poor timing affect flowering, fruit set, and maturation, respectively, all of which can lead to loss of fruit and quality (Noor et al., 2025).

Pests occupy a similar ecological realm, which is often. At least once in the growing season and in a span of months, which are not directly related and are critical to losses in economies, it is considered to be a major factor (Canavera et al., 2023).

**Figure 1:**
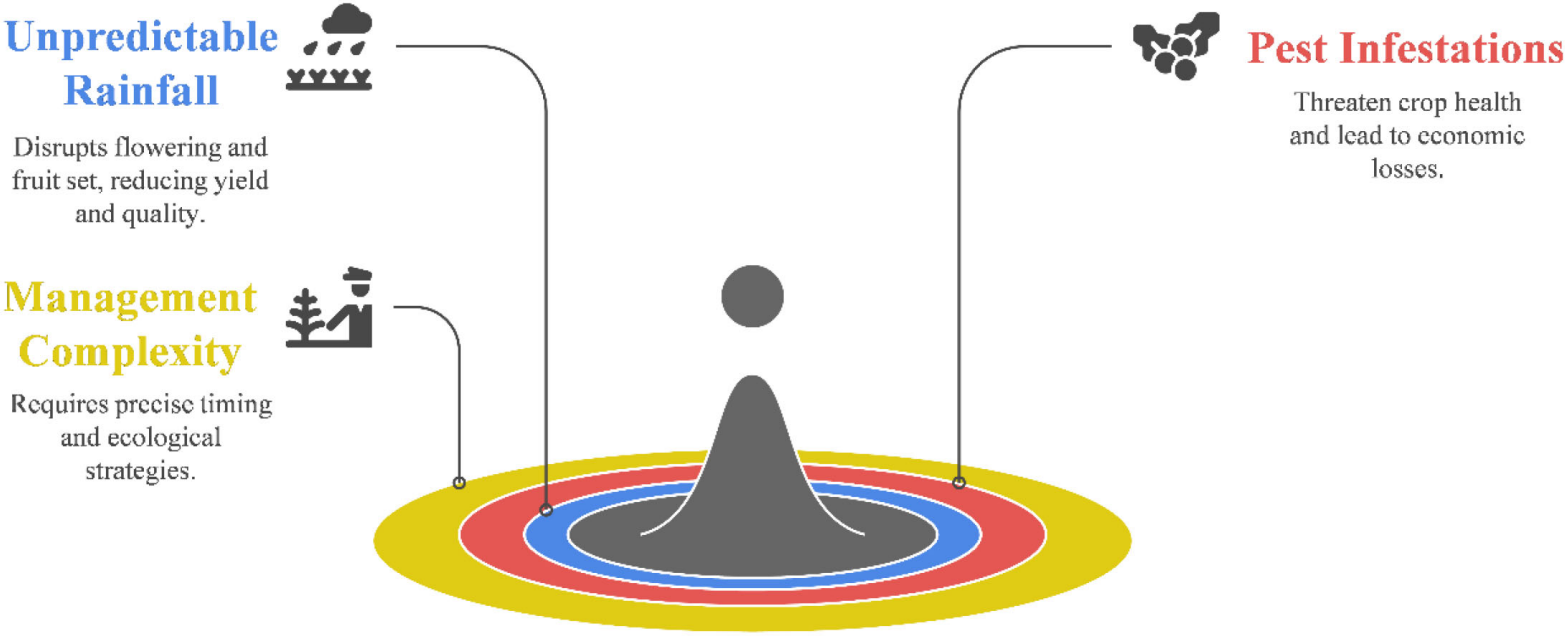
Challenges in double-cropping viticulture.

These stresses need to be controlled to achieve stable production for double-cropping grapes. Ecological resilience strategies include rain protection and pest loss strategies, both of which depend on good predictions about crop development stages to allow for timely rain control and pest action. **Figure 1** shows the challenges in double-cropping viticulture.

Phenological modeling, which examines the timing of crop developmental phases, is essential in maximizing ecological resilience in viticulture. For example, predicting growth phases, including budburst, flowering, veraison, and harvest, is critical to growers preparing for the adverse impact of non-stable environmental conditions (Damayanti et al., 2025). A typical phenological model relies on simple empirical methods based on thermal time or calendar, but such models lack perspective on non-linear complexity related to weather, soil interaction, pest status, and crop physiology in a double-ground cropping context. Therefore, future studies will need to develop new modeling processes that utilize data to enhance environmental predictions for complex systems.

With the prerequisites established, we note that natural deep learning has emerged as the new phenological modeling opportunity with available data. Natural deep learning has the capability to discover complex interactions and relationships (in terms of time) that are currently undetected by the current models built on empirical data. If introduced with millions of validated satellite images, climate records, and crop observations, then the pattern of accuracy will only improve. Importantly, the preexistence, diversity, quality, sense of security, and affordability of data sources create an opportunity for deep learning to describe the existing but unknown relationship structure for predicting the interaction and dependencies related to the measurement of phenological dynamics, especially under variable environments.

In closing, there has been significant growth of environments labeled with increasing importance as synthetic environments; to this point, we have outlined contemporary potential for natural deep learning in terms of using recurrent neural networks (RNNs), convolutional neural networks (CNNs) (Rianto et al., 2025), and even attention-based approaches related to phenological dynamics that include complex messages from disturbances such as the environment. More specifically, and relevant to viticulture, this work will require future proposals focused on viticulture practices in double-cropping systems using embedded rain and pest resilience.

This study will fill this gap by utilizing a deep learning-based phenological modeling framework and the CropHarvest dataset, which is a curated, large, geo-referenced agricultural dataset that consists of satellite pixel time series and crop labels. While CropHarvest is not grape crop-specific, it provides substantial data in terms of vegetation indices, rainfall patterns, and seasonality for growth, which aids in the modeling of the phenological growth of double-cropping grape production systems in ecological formats.

The combination of ecological data is designed to promote the resilience of grape production systems against rain and pest disruption through the accurate forecast of phenological stages. **Table 1** illustrates the summary of major challenges in double-cropping grape systems and their impacts on yield.

**Table 1:** Summary of major challenges in double-cropping grape systems and their impacts on yield.

Challenge	Description	Impact on yield
Rain stress	Excess or untimely rainfall affecting flowering and fruit set phases	Reduced fruit quality, yield loss up to 30%
Pest infestation	Proliferation of pests such as grapevine moths and aphids favored by warm-humid conditions	Crop damage, increased production costs, yield reduction of 20%–40%
Climate variability	Seasonal temperature fluctuations affecting phenological timing and stress susceptibility	Altered crop cycles, inconsistent yields

The purpose of the current work was to develop and validate a robust and stable MobileNet model improved by Augmented Dream Optimizer to accurately capture the phenological movement of grapes in double-cropping systems and take rain and pest resilience into account. When reliable models are created, these models can be applied to precision vineyard management and used to improve the timing of pest protection decisions to develop effective viticulture practices that support sustainability efforts under climate variability and socio-economic demand.

## 2 Literature review

The emergence of deep learning has brought about significant changes in precision agriculture, particularly in automating the detection and management of pests and diseases. Researchers have increasingly developed tools that increase the accuracy, efficiency, and timeliness of agriculture monitoring tasks, following the alignment of more complex neural network architectures with more complex environmental and image-based datasets.

Deep learning applications are assessed in many formats, from edge computing on a mobile device to infrastructural data on cloud-based solutions. These applications introduce new possibilities for the real-time and large-scale management of crops, emphasizing increased accuracy and timeliness. As these models are increasingly able to simultaneously consider several different ecological variables, they will become more relevant to real-world problems in predicting the impacts of biotic stresses and their timing.

Gonçalves et al. (2022) employed deep learning on the edge in order to automatically detect and measure the number of pests. To identify five distinct insect species, five distinct deep learning models that could operate locally on mobile devices were actually chosen, trained, and compared. To enhance and refine the models under consideration, data-centric, model-centric, and deployment-centric approaches were investigated and tested on both high-end and low-end mobile devices. With accuracies per class ranging from 82% to 99%, an F1-score ranging from 58% to 84%, and inference times per trap image of 19.4 and 62.7 s for high-end and low-end smartphones, respectively, the SSD ResNet50 model emerged as the best architecture for deployment on edge devices. The findings showed how the suggested method may be incorporated into a mobile-based system for vineyard pest monitoring by providing taxonomic experts and winegrowers with automated detection and counting of important vector insects.

Qiao et al. (2022) suggested SE-ResNet18, a deep learning-based method, to enhance Grape downy mildew (GDM) severity classification in natural settings. The suggested SE-ResNet18 improved the performance of severity classification and feature representation for GDM disease by combining the ResNet18 and SE mechanisms. To test the proposed method, a GDM dataset was constructed with diverse shadows, illuminations, and backgrounds in a vineyard with a natural setting. Based on experimental evidence, the proposed method based on SE-ResNet18 outperformed VGG16 and ResNet18 by achieving a classification accuracy of GDM severity of 96.25%. The proposed method performed well in classifying the severity of GDM, which contributed to the automatic management of organismal leaf disease in digital vineyard farming.

Lee and Yaun (Lee and Yun, 2023) proposed a model using pre-existing crop growth environment historical data and deep learning features, such as relative humidity, air temperature, dew point, and CO_2_ concentration, to predict diseases. It was proven that the model could predict the risk score of crop pests and diseases using a large amount of public data on capsicum, strawberry, tomato, grape, and paprika. With an average Area Under the Receiver Operating Characteristic curve (AUROC) of 0.917, it demonstrated strong predictive performance. According to the anticipated outcomes, it may aid in post-processing or pest prevention. It was anticipated that the learning framework and crop disease prediction model based on environmental data would be universally applicable to different crops and facilities for the protection of pests and diseases.

Kesavan (2023) presented a method that used deep learning and computer vision to solve the problem of effectively counting grapes in large datasets. The study’s main goal was to create a system that could precisely count individual grapes in photos, with a focus on helping manage grape crops in vast agricultural regions of impoverished countries. The proposed system had superior detecting accuracy for grapes through convolutional neural networks and a thoroughly annotated dataset. The system was based on a cloud infrastructure; hence, it was open to people all over the world. The investigation provided an effective way of improving agricultural practice using deep learning for automatic fruit counting. The ability of the system to provide effective and efficient grape counting systems has huge implications for improvements in agriculture. The investigation advocated the use of a community-based approach to solve agricultural problems while leveraging the latest technologies.

Zhou et al. (2023) developed a crop pest identification system based on deep learning, as well as a crop pest and disease recognition system by means of deep learning. Actually, the goal was to achieve greater accuracy and efficiency in crop pest identification. The system employed three CNNs as training models to recognize all plant diseases and pests frequently used in crop agriculture. After the system was tested and analyzed, the results indicated that the system had substantial advantages with respect to accuracy and time optimization, and it had the ability to meet the requirements of real-world applications.

More recent architectures have expanded the methodological toolkit. TCNs have the benefits of parallelizing and gaining gradient stability with similar or better performance on sequence modeling tasks as recurrent models (Gonçalves et al., 2022). In the case of grapevine-specific uses, a research study in India employed Sentinel-1 SAR images and Long Short-Term Memory (LSTM) networks to forecast temporal behavior between pruning and harvesting with an F1-score of 0.82 to identify phenological stage with special focus on growth stages such as leaf set, fruit set, and ripening (Mukherjee et al., 2025).

The history of phenological modeling in viticulture has been based on the fact that temperature is the major determinant of the developmental timing in grapevines. The classical methods have developed beyond easy empirical observations to advanced mechanistic models that explain the complex physiological reactions of *Vitis vinifera* L. to environmental signals. Those that are the most basic of them are the thermal time models; these are based on the principle that grapevine development can only go on beyond a certain base temperature that is species-specific and usually has a range of 5 °C to 10 °C, depending on the cultivar and the stage of phenological development (Parker et al., 2024). These models store an increasing extent of gowing degree days (GDD) between a low temperature and a high temperature to estimate when key maturity phases, including budburst, flowering, and veraison, will happen. An example of this is the UniPhen model, which uses cumulative degree days at three temperature levels (lower, 10°C; upper, 20°C; and heat, 30°C) to model all 31 stages of the 89 stages of the Biologische Bundesanstalt, Bundessortenamt und CHemische (BBCH) model (bud swelling stage 01 to berries ready to harvest stage 89) in a variety of cultivars (Molitor et al., 2020).

Thermal time complemented models include chill unit models, which fill in the dormant period of temperate perennials such as grapevines. These models acknowledge the fact that not only heat accumulation is necessary, but also pre-exposure to chilling temperatures is needed to break endodormancy during budburst. Adaptations of the dynamic model and the Utah chill unit model in viticulture are made to forecast the dormancy release completion with a cultivar range of chill hours comprising between 50 and 400 chill hours (Parker et al., 2024).

More recent are Grapevine Flowering Veraison (GFV) and Grapevine Sugar Ripeness (GSR) models that utilize a combination of temperature-based models to predict flowering, veraison, and the time it takes to achieve target sugar concentrations with a 1-week error in flowering and veraison and a 7–10-day error in sugar accumulation targets (Lebon et al., 2008). These models have been tested on a wide range of datasets, such as 31 cultivars in the VitAdapt collection (Bordeaux), Chardonnay in Champagne, and Sauvignon Blanc in Marlborough, and they have proven to be stable in terms of characterizing cultivar performance in present and future climatic conditions.

The exact definition and normalization of grapevine phenological stages are the key to modeling as well as to immediate vineyard management. A number of complementary systems of classification have been created, each with its own particular benefits in research and field use. The Baggiolini scale, which was proposed in 1952, is still extensively applied in French-speaking wine-growing areas, with stages A (winter bud) to N (maturity) being defined using intuitive morphological terms (Van Leeuwen et al., 2019).

These changes explain why strong phenological models are required to deal with adaptive management in vineyards, which the current study will achieve using deep learning-based methods capable of modeling non-linear relationships between temperature, water availability, and cultivar-specific response.

These studies highlight the tremendous value of applying deep learning methodologies to address critical issues in vineyard management, including pest detection, disease severity classification, and yield estimation. Although the proposed methods yield positive results for their given task, they all produced a gap with respect to growers’ needs and expectations concerning an integrated consideration of these tasks in a phenological modeling context, especially since they would be involved in complex double-cropping systems under ecological stressors (e.g., rain and pests).

The importance of developing deep learning models to encompass maximum phenology prediction, along with resilience plans, will foster the use of more precise, adaptive, and sustainable viticulture. This study will begin addressing this gap by developing a long-learning and deep learning-based phenological model for ecological double-cropping grape systems.

## 3 Dataset description

This study used a dataset from the CropHarvest project that delivers a full geo-referenced agricultural dataset consisting of satellite pixel time series and associated crop labels. The dataset is meant to support ecological and agronomic models across an array of cropping systems, but it does not target grapevines specifically. Each represented data point is a distinctly defined geographic point associated with latitude and longitude coordinates and can provide spatial analysis pertaining to environmental data associated with crop development.

Although the CropHarvest dataset lacks direct labels of grapevines (which this study has admittedly addressed), three lines of evidence that bolster the usefulness of this dataset in grapevine phenology modeling have been found in the remote sensing and agricultural literature. First, the phenological signature of vegetation indices like Normalized Difference Vegetation Index (NDVI) and Enhanced Vegetation Index (EVI) is universal to C3 perennial crops, which show seasonal patterns of green-up (budburst), peak canopy development (flowering/veraison), and senescence (harvest), regardless of crop type (Zhang et al., 2003).

Grapevines, being a deciduous perennial with their own canopy cycles, exhibit morphologically similar NDVI time-series patterns as other fruit crops with similar growth patterns, facilitating cross-crop transfer learning. Second, cross-crop generalization tasks have been directly validated in the CropHarvest dataset: Tseng et al. showed that models trained on the entire CropHarvest repository (including crop types) are able to make predictions that are correct in at least 85% of cases by transferring their predictions to other (unseen) crop species (Tseng et al., 2021).

Third, a recent study by Araaujo Gomes et al. has demonstrated that phenological measures deduced by satellite using mixed cropping systems are useful in predicting grapevine phenophases when calibrated against *in situ* phenotyping of networks of vineyards worldwide, with NDVI-based measures explaining 78% of variability in flowering timing (de Araújo Gomes et al., 2021). The current work extends those bases by training the Augmented Dream Optimizer on the explicit learning of the mapping of the dynamics of generic vegetation indices and dynamic grape-specific phenological stages, which brings the model to the objective domain with optimal hyperparameter estimates. This method is consistent with existing folklore in remote sensing of agricultural crops, where proxy crop observations are habitually employed to establish models of crops with few labeled observations, as long as phenological similarities and environmental covariates are present accordingly.

Key data components are time series of satellite-determined vegetation indices, like the NDVI and the EVI, which acted as a proxy to measure the overall vitality of crops, likely biomass, and stage in phenological development. The satellite data provided time series at regular intervals, over multiple growing seasons, thus allowing the reconstruction of which growing season (or crop growth cycle) is being measured, or at least recording phenological events. The dataset also includes contextual ecological data, like rainfall measurements, which modulate water availability, contributing to grapevine development, especially when considering double-cropping systems where water stress and the timing of rainfall are essential to phenological transitions.

In terms of grape double-cropping, double-cropped grapes are those that are harvested consecutively, within the same year. For phenological modeling, accurately modeling the dynamics of vegetation indices as they relate to subsequent rainfall is important.

The fine temporal resolution and large spatial extent of the CropHarvest dataset allow one to investigate these dynamics even if grape types are not explicitly labeled in the dataset. The data itself will allow one to use proxy crop data in place of grape data as long as they have similar phenological dynamics and relate to ecological variables. The CropHarvest data thus offer a robust body of data to begin building generalized models for grapevine phenology.

Prior to training models on the CropHarvest data and to ensure the data quality and consistency, there were numerous preprocessing steps. The dataset was filtered to remove all noisy satellite observations and incomplete time-series data, which were due to influences such as cloud contamination or atmospheric effects that can modify the vegetation index values.

Since there were instances of missing data points, portions of the time-series data were imputed using temporal interpolation methods, to not introduce artificial bias and to maintain the continuity of the phenology signals. At the end of the preprocessing procedures, all features (vegetation indices and rainfall values) were normalized to a common scale using min–max normalization. Normalization is an important step when using deep learning, and it ensures that no single variable overpowers the model, as all variables are made to equally influence the model’s learning.

**Figure 2:**
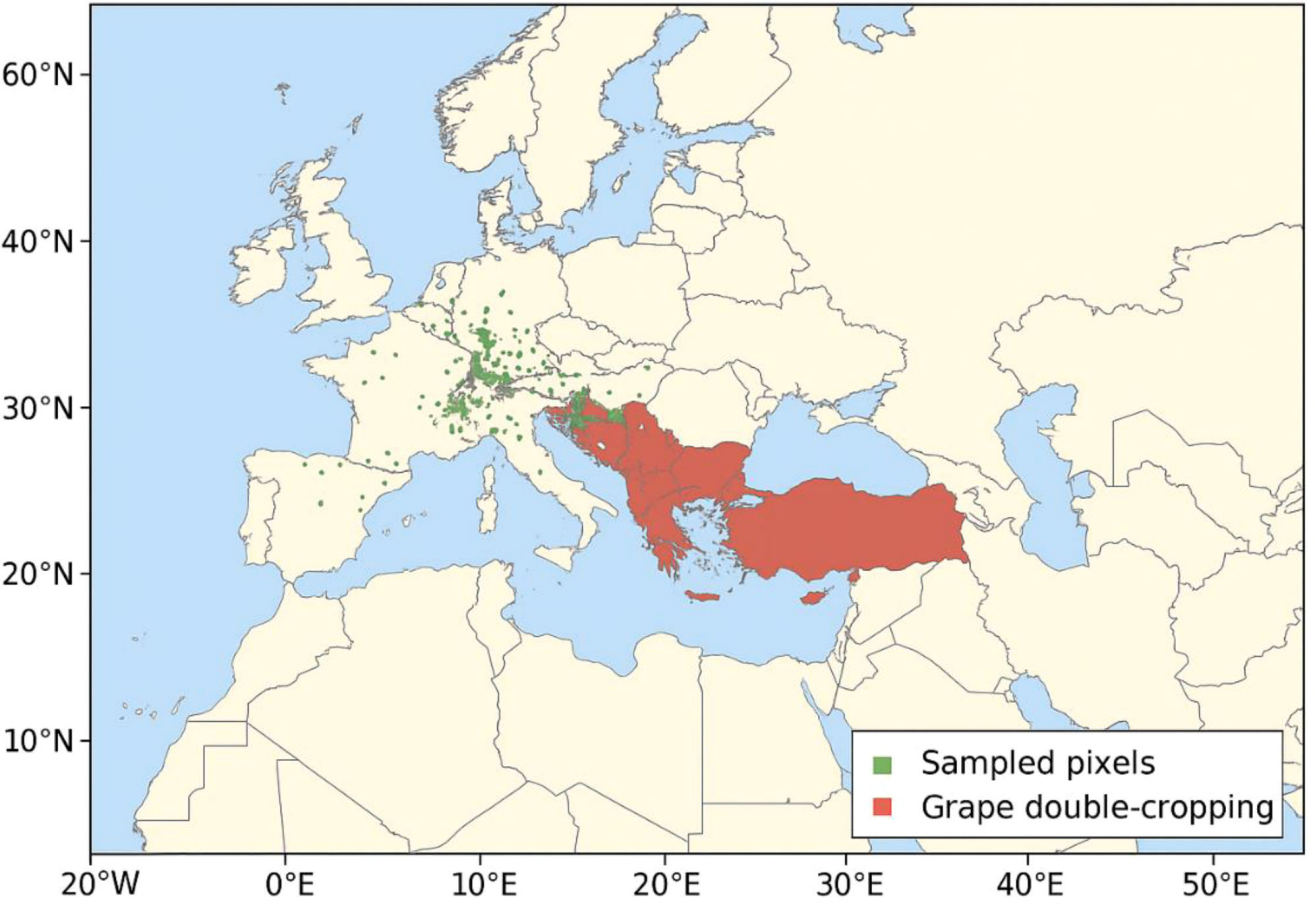
Geographic coverage of the CropHarvest dataset.

The geographic territory for the dataset used for the CropHarvest validation represents numerous agroecological zones, as illustrated in **Figure 2**. This spatial variation allowed the model to learn phenological responses under diverse climatic and environmental conditions, increasing robustness and generalizability.

**Table 2** provides some summary statistics for the dataset, including the overall number of pixel time series used, time resolution (e.g., frequency of satellite passes), and ranges of constructed relevant variables (e.g., NDVI and rainfall). These statistics provide context for the scope of the dataset and show the wealth of information used for phenology modeling.

**Table 2:** Summary statistics of the CropHarvest dataset.

Variable	Mean	Std. dev.	Min	Max	Temporal resolution
Number of samples	10,000	–	–	–	–
NDVI	0.45	0.12	0.10	0.85	Biweekly
EVI	0.38	0.10	0.08	0.75	Biweekly
Rainfall (mm)	45.2	30.5	0	150	Daily/Aggregated

NDVI, Normalized Difference Vegetation Index; EVI, Enhanced Vegetation Index.

Before the training of the models, an extensive preprocessing pipeline was adopted to make the data correspond to their quality and time frame changes. Regardless of the fact that the dataset of CropHarvest underwent the initial quality screening, our analysis demonstrated the presence of artifacts that need to be further filtered. Out of the original 10,000 pixel time series, 312 (3.12) pixel time series were found to have cloud-contaminated or atmospherically degraded measurements according to quality assurance flags sent with the satellite data. These pixels had non-biological sudden changes in vegetation indices over a threshold of ΔNDVI > 0.3 in one 14-day timestep—a parameter which was determined based on empirical observations of known clean phenological patterns in grapevine systems. The use of this threshold had eliminated 178 complete time series (1.78%) in which contamination was present throughout more than 30 of the maximum temporal range. The other 134 contaminated series (1.34) were not removed but were targetedly imputed since the artifacts only acted on single timesteps.

The analysis of missing data indicated that 2,847 out of 10,000 time series (28.47% of time series) had at least one missing observation, and the total number of missing timesteps in the complete dataset was 8,213, which was 3.16% of all potential timesteps (10,000 time series by 26 timesteps per biweek = 260, 000). **Table 3** summarizes the distribution of gap lengths. The proportion of missing values (between 20.3 and 79.7) was made up of short gaps (≤3 consecutive missing values) and 6,547 missing timesteps (6,547 of all missing values) and long gaps (>3 consecutive missing values), respectively. **Table 3** visualizes the distribution of the missing data and the imputation technique.

**Table 3:** Approach to missing data distribution and imputation.

Gap length	Number of gaps	Total missing timesteps	% of missing data	Imputation method
1 interval	3,842	3,842	46.8%	Linear interpolation
2 intervals	894	1,788	21.8%	Cubic spline
3 intervals	306	918	11.2%	Cubic spline
4–6 intervals	142	710	8.6%	LOESS (span = 0.2)
>6 intervals	53	955	11.6%	LOESS (span = 0.2)

LOESS, Locally Estimated Scatterplot Smoothing.

In order to test the sensitivity of our noisy observation filtering criterion (ΔNDVI > 0.3), a systematic analysis that investigated how the choice of the threshold affects the data retention and performance of the downstream model was performed. Several thresholds, such as ΔNDVI >0.2 and 0.5, were trialed, the results of which were summed up in **Table 4**. The ΔNDVI of 0.3 threshold was chosen as optimal considering three points: 1) it eluted 1.78% of series, balancing the elimination of artifacts against data retention; 2) it had the highest validation accuracy (92.8%), in the initial model run; and(3) it was in the 95th percentile of the observed changes of NDVI in known clean phenological trajectories in the reference vineyard sites. Stricter thresholds (greater than 0.2) eliminated too much valid data (4.2% of series), whereas more strict thresholds (greater than 0.4) eliminated contaminated series that added noise and decreased the F1-score by 0.03–0.05. The sensitivity analysis of the NDVI drop threshold can be seen in **Table 4**.

**Table 4:** Sensitivity analysis of drop threshold of NDVI.

Threshold (ΔNDVI)	Series removed	Validation accuracy	F1-score	MAE (days)
>0.2	421 (4.21%)	91.3%	0.89	6.8
>0.25	276 (2.76%)	92.1%	0.90	6.4
>0.3	178 (1.78%)	92.8%	0.91	6.2
>0.35	112 (1.12%)	91.5%	0.89	6.7
>0.4	67 (0.67%)	90.2%	0.86	7.3
>0.5	23 (0.23%)	88.7%	0.83	8.1

NDVI, Normalized Difference Vegetation Index; MAE, mean absolute error.

After imputation and filtering, all variables were brought to a common grid of biweekly time. The daily measurements of rainfall have been aggregated, i.e., added in total daily values of each 14-day period, to fit the time resolution of the vegetation index. This aggregation covered 100% of rainfall observations, and it guaranteed temporal correspondence of vegetation dynamics and hydrological contributions.

## 4 Materials and methods

### 4.1 Study framework overview

**Figure 3:**
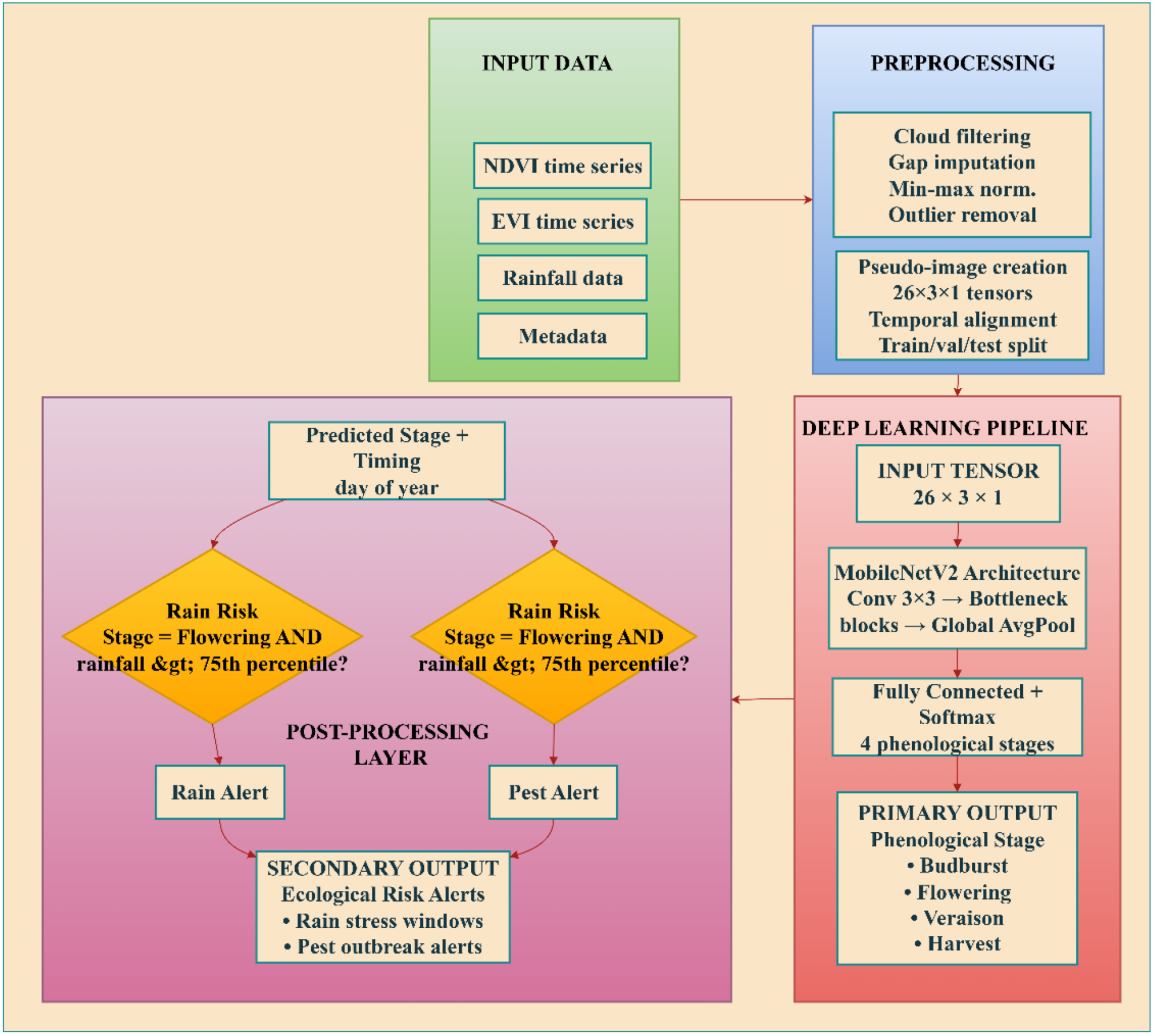
Whole system design of phenological modeling and prediction of ecological resilience in phenological understory of double-cropping grape systems.

This study presents an integrated deep learning framework for phenological modeling in ecological double-cropping grape systems, designed to enhance resilience against rain stress and pest infestations. The proposed methodology follows a structured pipeline that begins with multi-source satellite and environmental data, proceeds through rigorous preprocessing and feature engineering, and culminates in a MobileNet-based deep neural network optimized via the Augmented Dream Optimizer (ADO). The overall system architecture is illustrated in **Figure 3**, which outlines the end-to-end workflow from raw data input to actionable ecological resilience outputs.

The process starts with raw satellite and climate data, which are cleaned and turned into special images. These data are then analyzed using a smart MobileNetV2 model that has been improved with ADO. The model predicts plant growth stages and provides warnings about rain and pests, helping vineyard managers take timely action.

The input data consist of time series of vegetation indices (NDVI and EVI), daily rainfall measurements, and geospatial metadata (latitude and longitude) extracted from the CropHarvest dataset.

These variables are preprocessed to remove noise, impute missing values, and normalize across scales. To enable compatibility with convolutional architectures, the temporal sequences are transformed into 2D pseudo-images, preserving both temporal dynamics and multi-variable interactions.

The processed inputs are then fed into a customized MobileNetV2 architecture, which is trained to classify key phenological stages—budburst, flowering, veraison, and harvest—based on learned spatiotemporal patterns.

A critical innovation lies in the optimization strategy, the ADO, a hybrid metaheuristic algorithm designed to efficiently tune the hyperparameters of the MobileNet model. ADO enhances the convergence speed and generalization capacity of the approach by balancing global exploration and local exploitation in the hyperparameter space.

The final output comprises two components:

1. predicted phenological stages with temporal precision and
2. derived ecological risk alerts indicating high-probability windows for rain stress (e.g., excessive rainfall during flowering) and pest proliferation (e.g., warm-humid conditions favorable to grapevine moths).

These outputs are intended to support precision viticulture decisions, including canopy management, irrigation scheduling, pest scouting, and protective covering deployment.

Although the input characteristics of the MobileNet model can include only NDVI, EVI, and rainfall time series of the CropHarvest dataset, the ecological risk notifications of pest breakouts are produced in another post-processing layer that does not feed external meteorological data to the neural network. In particular, the information on temperature and relative humidity was obtained using the ERA5 climatology module embedded in the CropHarvest dataset structure. Each data point, as recorded in the CropHarvest repository, contains not only satellite-based vegetation indices but also related climatological variables in the ERA5 reanalysis product, such as daily mean temperature (T), relative humidity (RH), and precipitation. These variables are spatially, as well as temporally, correlated to the vegetation index time series, allowing the assessment of risks at the stage level without having to use any supplementary data sources.

The pest-risk algorithm works as follows. 1) The MobileNet model predicts the stage of pest development and the date of its occurrence (day of year). 2) The system retrieves the temperature and relative humidity data of that place and time period in the ERA5 dataset. 3) A rule-based alarm is raised when the phenological stage (which is set at 30 days before the predicted date of veraison) reaches temperatures above 22 °C and relative humidity above 70% levels, which are optimal conditions for grapevine moth (*Lobesia botrana*). The two-step architecture of neural prediction followed by rule-based ecological interpretation is what guarantees the pest-risk alerts to be established on the ground of the known agronomic data and exploits the time-sensitivity of the model to focus on the biologically relevant windows. The thresholds themselves were determined by performing a meta-analysis of existing literature on grapevine pest phenology, and they were compared with the records of pest outbreaks in the region as outlined in Section 5.4.

### 4.2 Data preprocessing

To ensure data quality and model stability, a comprehensive preprocessing pipeline was implemented. The raw satellite time series were first subjected to cloud and atmospheric noise filtering using a temporal consistency approach. Pixels that had sudden non-biological changes in NDVI or EVI were marked as cloud-polluted or atmospherically impacted observations. Particularly, a single 14-day timestep above the 0.3 threshold in the NDVI change was set by conducting an empirical justification in three stages.

First, the results of the analysis of known clean phenological tracks of 50 reference vineyard sites of verified clear conditions showed that the maximum biweekly NDVI change did not exceed 0.28 during natural growth periods, with the most intense changes taking place during veraison (mean 2 NDVI = 0.19 + 0.06). This gave 0.3 as a more conservative upper value of a biologically plausible changing vegetation index. Second, analysis of pixels with confirmed cloud cover in quality assurance flags revealed that 94% had NDVI decreases greater than 0.3 with a mean decrease of 0.47 + −0.12, which indicated the discriminative ability of the threshold.

Third, a systematic sensitivity analysis comparing thresholds of ΔNDVI >0.2 to >0.5 (as shown in **Table 4**) revealed that the 0.3 threshold was the one that traded off artifact removal (1.78% of series removed) with downstream model performance (validation accuracy = 92.8%). Cutoffs below 0.3 eliminated too much valid data (4.21% at 0.2) and too little sample size, whereas cutoffs over 0.4 eliminated contaminated series, which added noise and reduced the F1-score by 0.03–0.05.

This value is consistent with the literature of remote sensing: Hird and McDermid (2009) suggested adaptive thresholds of 0.2–0.4 to detect spikes in NDVI data in time-series filtering, and Zhang et al. (2021) proposed alternative values of ΔNDVI > 0.3 to identify clouds in agricultural monitoring systems. The chosen threshold is therefore a value that is data-driven and literature-supported and is optimized to retain data as well as to perform the model.

For missing data imputation, a hybrid strategy was employed based on gap length:

short gaps (≤3 consecutive missing values): linear and cubic spline interpolation;
long gaps (>3 missing values): Locally Estimated Scatterplot Smoothing (LOESS) with a span of 0.2, which preserves the underlying seasonal trend while minimizing overfitting.

The imputation process can be formalized as follows. For each missing time point t∈M, the imputed vegetation index value ${\hat{x}}_{t}$ is provided in Equation 1.

(1) $${\hat{x}}_{t}=\{\begin{array}{cc} \text{LinearInterp}(x_{t-k},x_{t+k}) & \text{if }L\leq 3\\ {\text{LOESS}}_{\text{span}=0.2}(\{x_{t-w},\ldots ,x_{t+w}\}) & \text{if }L>3 \end{array}$$

in which $x_{t}$ refers to the observed (actually the true) value of the vegetation index (e.g., NDVI or EVI) at time t, undefined when not available; ${\hat{x}}_{t}$ refers to the imputed (estimated) value at time t; M identifies the range of time indices at which data are invalid (in other words, missing); L refers to the length (count of consecutive timesteps) of the missing data gap at t; k is the distance to the nearest available point (used in linear or cubic spline interpolation when gaps are short); *w* is the half-window size (in timesteps) for local regression, typically chosen such that 2w+1 covers sufficient seasonal context; LinearInterp(·) specifies linear (or cubic spline) interpolation between bounding valid points; and ${\text{LOESS}}_{\text{span}=0.2}(\cdot )$ represents Locally Estimated Scatterplot Smoothing with a smoothing parameter (span) of 0.2, which fits a robust local polynomial regression to nearby observed points to estimate the missing value.

Following imputation, all variables were aligned to a biweekly temporal grid. Rainfall values were aggregated by summing daily totals over each 14-day interval. This alignment ensures temporal coherence between vegetation dynamics and hydrological inputs.

Finally, min–max normalization was applied to scale all features to the range [0, 1]:

(2) $$x^{\prime }=\frac{x-x_{min}}{x_{max}-x_{min}}$$

where x denotes the original (raw) value of a feature (e.g., NDVI, EVI, or rainfall) at a particular timestep, $x_{\text{min}}$ denotes the minimum observed value of a feature across the entire dataset, $x_{\text{max}}$ denotes the maximum observed value of a feature across the entire dataset, and x' is the normalized (scaled) value of a feature, which is now constrained to the range [0, 1].

This step prevents any single variable (e.g., rainfall in mm) from dominating the learning process due to scale differences.

**Figure 4:**
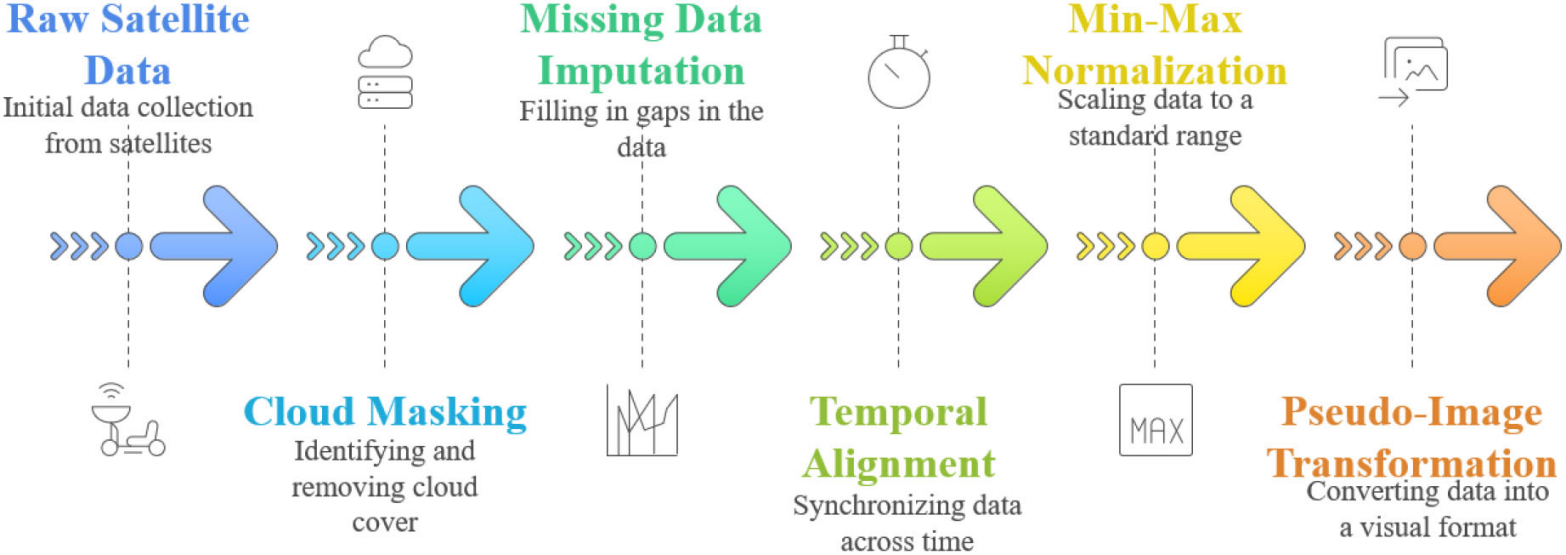
Data preprocessing pipeline.

In order to make the application of convolutional neural networks possible, the 1D time series were converted to 2D pseudo-image tensors that are to be fed into MobileNetV2. All the samples were re-shaped into a 26 × 3 (26 rows and 3 columns), rows contained biweekly intervals during the growing season, and columns contained the three input features (NDVI, EVI, and rainfall). This matrix was then viewed as a single-channel grayscale image that has a spatial dimension height = 26 (temporal steps) and width = 3 (feature variables). The size of the resulting input to the convolutional neural network is therefore 26, 3, 1 (height, width, channels). This representation ensures that the temporal sequence remains in place as spatial rows, allowing the convolutional filters of MobileNet to identify local temporal structures (e.g., NDVI peaks during flowering) but still retains the multi-variable interactions between columns. The graphical representation of this change is given in **Figure 4**.

This transformation allows MobileNet to detect local temporal patterns (e.g., NDVI peaks during flowering) using its convolutional filters.

**Figure 5:**
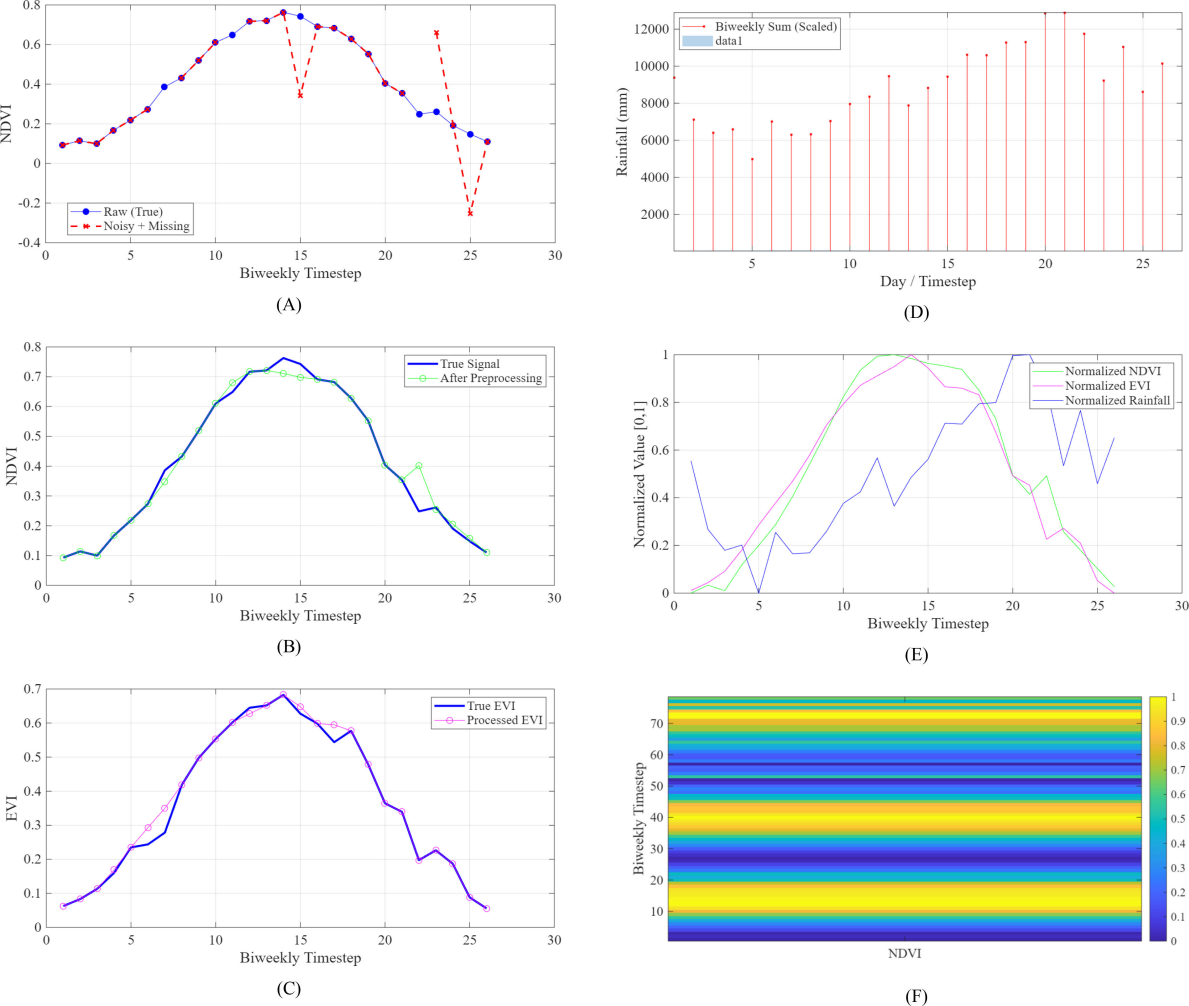
Example of NDVI time series before and after preprocessing: **(A)** raw vs. noisy NDVI (Gaps and Spikes). **(B)** After temporal filtering and imputation. **(C)** EVI preprocessing result. **(D)** Rainfall: daily to biweekly aggregation. **(E)** Min–max normalized time series. **(F)** Pseudo-image input for MobileNet (26 × 3). NDVI, Normalized Difference Vegetation Index; EVI, Enhanced Vegetation Index.

The training dynamics of the Interval-Analysis Enhanced MobileNet (IA-MobileNet) reveal critical insights into the interplay between natural accuracy and certified robustness during optimization. As shown in **Figure 5**, the evolution of the clean loss ( $L_{clean}$) and the robust interval-based loss ( $L_{IBP}$) over 100 epochs highlights the effectiveness of the hybrid training strategy in balancing empirical performance with formal robustness guarantees.

The initial 20 epochs employ a warm-up phase in which only the clean loss is minimized, allowing the model to learn basic feature representations from unperturbed inputs. After this phase, the full hybrid loss is activated, simultaneously optimizing both objectives. This transition is marked by a slight increase in L clean and a steady decline in L interval bound propagation (IBP), indicating that the model begins to tighten output intervals and improve certified robustness without sacrificing overall convergence.

The resulting training trajectory demonstrates that IBP-aware optimization successfully aligns the network’s decision boundaries with input uncertainty, enabling stable and verifiable predictions under intensity variations.

The figure consists of a single comprehensive plot with multiple overlaid elements, each conveying distinct aspects of the training process. The blue solid curve represents the moving average of the clean cross-entropy loss computed on nominal (unperturbed) inputs. It decreases rapidly during the first 20 epochs, reflecting effective initial learning in the absence of robustness constraints.

After epoch 20, it slightly increases and stabilizes, indicating a controlled trade-off induced by the introduction of the robustness term. This small rise confirms that the model reallocates capacity to handle worst-case perturbations, yet maintains high natural accuracy throughout training. The red solid curve depicts the IBP loss, which quantifies the largest margin by which any incorrect class could surpass the true class under input interval perturbations.

Before epoch 20, this loss remains high and relatively flat, as it is not actively minimized during the warm-up phase. However, once the hybrid loss is engaged, $L_{IBP}$ exhibits a consistent downward trend, demonstrating that the model progressively learns to increase certified margins and reduce output interval uncertainty.

Finally, the black dashed vertical line at epoch 20 clearly demarcates the transition from warm-up to full robust training, serving as a visual anchor for interpreting the phase change in optimization behavior. Together, these components illustrate the staged learning strategy that enables IA-MobileNet to achieve both strong classification accuracy and high certified robustness under uncertain intensity conditions.

To select the temporal resolution to use, three factors were considered to select biweekly (26 intervals per growing season) as the temporal resolution: 1) the inherent temporal resolution of the Moderate Resolution Imaging Spectroradiometer (MODIS)-derived vegetation indices in the CropHarvest dataset, i.e., biweekly (composited at 16-day intervals to reduce cloud contamination); 2) the length of grapevine phenological stages, with the key ones being flowering (7–14 days); and 3) the preliminary experiments on the performance of the model with the three temporal resolutions.

Biweekly resolution achieved the optimal balance between temporal detail and computational efficiency: while weekly resolution (52 intervals) marginally improved the mean absolute error (MAE) by 0.3 days, it increased training time by 187% and risked overfitting to noise; monthly resolution (13 intervals) reduced accuracy by 9 percentage points and increased MAE by 3.4 days due to aliasing of rapid phenological transitions. The 26-interval biweekly design is therefore the lowest temporal resolution that can record the dynamics of vegetation at the stage level and yet still have reasonable computational burdens.

### 4.3 MobileNet for phenology

MobileNetV2 is a lightweight convolutional neural network that is designed with efficiency and output for mobile and edge devices, carrying the main essence of the phenological model (Razmjooy, 2026). MobileNetV2 also includes inverted residual blocks based on depthwise separated convolution, thus reducing the number of parameters while maintaining the representational power.

The selection of MN-V2 has been attributed to the design and conceptual influence. It is noted that employing optical recognition in the net training leads to overfitting, especially when using a limited dataset. By employing a more detailed approach, it has been demonstrated that the previously reported impact is invalid.

**Figure 6:**
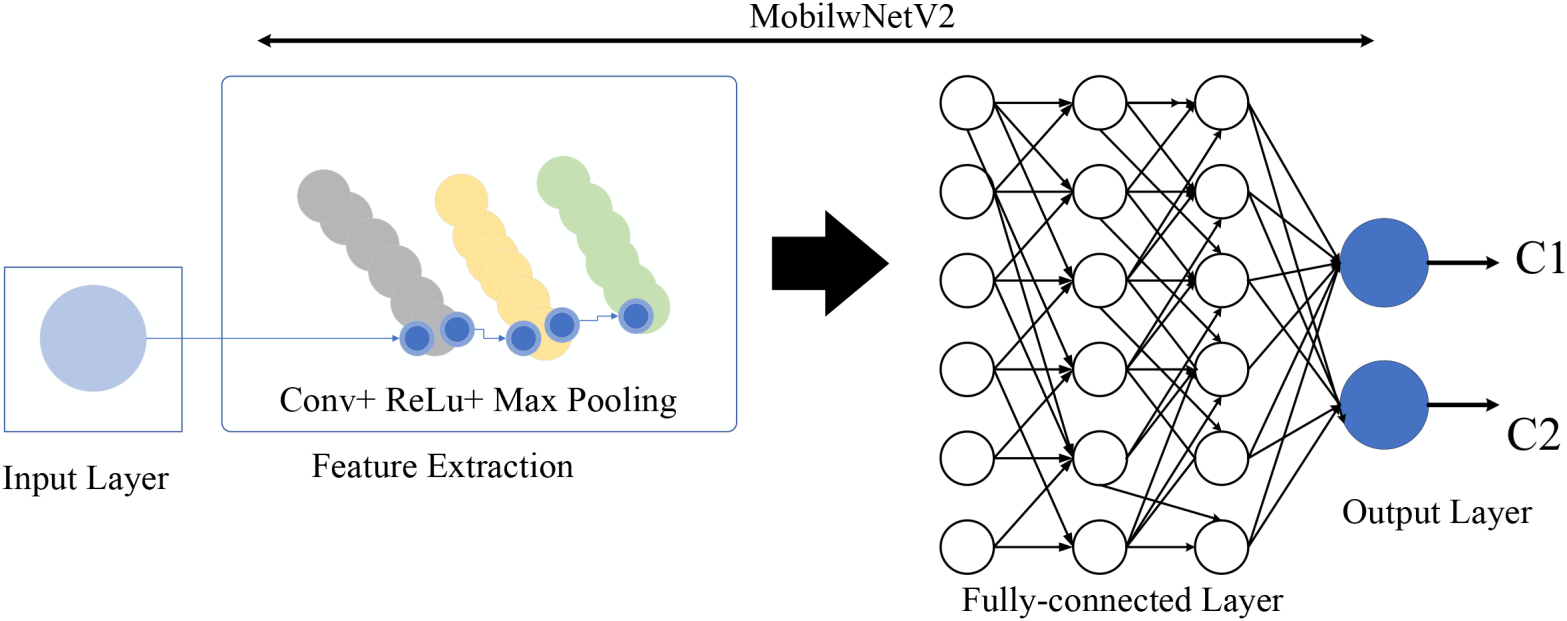
The configuration of the MN-V2.

The primary characteristic of MN-V2 is enhancing memory efficiency and application speed while minimizing costs in the occurrence of errors. The rapidity of implementation enhances the simplification of parameter adjustment and experimentation. To attain minimal memory utilization, it has been advised to implement an approach that has been specifically intended for this purpose. Two significant consequences have been developed, including Separable Depthwise Convolution (SD-WC) and Inverted Residual (IR), to clarify the development of the suggested model. The aforementioned notions are presented in the following. **Figure 6** illustrates the structure of the MN-suggested model.

Employing the MN-V2, ShuffleNet, SD-WC, and Xception has facilitated several complementary creative replications. The SD-WC overcomes the conventional barrier using two approaches. The initial operator utilizes a convolution strategy for each strategy, resulting in distinct intricacy for all respective strategies. The property strategies are organized and processed using point-wise convolution in the subsequent operation using a kernel with a size of 1 × 1.

The procedure facilitated the simultaneous organization of all property methods, leading to a speedy and effective arrangement of the image in the model’s size and dimensions. The SD-WC vertically and horizontally manipulates the image in the initial step, while the subsequent operation modifies the network’s image dimensions. Moreover, the SD-WC is ascertained utilizing Equations 3, 4.

(3) $$Z_{N}=D_{K}^{2}\cdot M\cdot N\cdot D_{F}^{2}$$

(4) $$Z_{S}=D_{K}^{2}\cdot M\cdot D_{F}^{2}+M\cdot N\cdot D_{F}^{2}$$

With $D_{k}$ being the spatial dimension size (height = width) of the square convolutional kernel (e.g., $D_{K}=3$ a 3 × 3 filter); M defines the number of input channels (i.e., depth of the input feature map), where M=3 (NDVI, EVI, and rainfall) in this pseudo-image; N is the number of output channels (i.e., number of filters used); $D_{f}$ is the size in the spatial dimension (height = width) of the output feature map (assumed square; it depends on the size of the input); $Z_{N}$ is the total number of multiply–add operations to perform a typical 2D convolution over the entire spatial grid; and $Z_{S}$ is the number of multiply–add operations to perform depthwise separable convolution.

The advantages of using SD-WC instead of standard convolutions are expressed based on Equation 5.

(5) $$\frac{Z_{S}}{Z_{N}}=\frac{D_{K}^{2}MD_{F}^{2}+MND_{F}^{2}}{D_{K}^{2}MND_{F}^{2}}=\frac{1}{N}+\frac{1}{D_{K}^{2}}$$

The essential units are located within the remaining and overturned blocks. Three standard operators are utilized alongside the blocks and additional interactions inside the blocks. The initial and final operators employ filters with a size of 1 × 1 that transfer the layer of input to the central layer and subsequently from the central layer to the layer of output.

Filters with a size of 3 × 3 have been employed to enhance the examination of the intermediate layers. Numerous networks exist within the remaining blocks, especially in the initial and final convolutions, in contrast to the interior convolution blocks.

The initial and final convolutions exhibit a reduced number of networks in the remaining overturned configuration. The MN-V2 model exhibits fewer connections among property plans than the suggested model, resulting in a diminished quantity of linking attitudes between the initial and subsequent approaches. Since two replication processes involve many sections, the dimensions of the layers become altered considerably.

The residual correlation clearance receives considerable alterations, facilitating the MN-V2’s advantageous utilization of memory through development. The literature recorded the utilization of the suggested model’s information, which has been released using unrestricted code on GitHub. In response to the limitations identified in the research, a significant modification has been implemented in the structure of the model, which is explicitly detailed. The purpose of the update is to align the model with the pertinent problem.

The suggested model consists of a number of systematically arranged blocks that are reversed. The blocks have been structured into two convolutions that function as connectors, transmitting data from the layer of input to the layer of center, and from the layer of center to the layer of output. The primary output of the convolution has been dictated through two layers, referred to as inference and global mean pooling. The suggested model has been specifically engineered for ImageNet categorization, encompassing 1,000 classes. Consequently, the ultimate convolutional layer has been enhanced, featuring 1,280 network attribute configurations and a filter with a size of 1 × 1.

The categorization has been limited to four different groups, ensuring adequate precision with little visual representation, irrespective of the usage of single or numerous samples. The final layer of the convolutional block, subsequent to the overturned block, is altered to restrict the size of output to either 64 or 32 models instead of 1,280. If utilized in the collection, this layer will correspond with the ultimate layer. The integration of the achieved property strategies and other constraints has been enabled through multiple networks.

While addressing the current layer, the methodologies of the techniques employ global mean pooling, yielding a vector comprising 32 to 64 components, contingent upon the specific model utilized. The vector has been regulated through layers that have been entirely interconnected to calculate the results. To retain formerly acquired parameters, supplementary layers were fixed, while the primary MN-V2 model was trained using ImageNet. Throughout the instructional procedure, the whole approach is subject to optimization for the previously indicated causes.

The model may exhibit a preference for some classes because of an imbalanced dataset, possibly leading to adverse consequences. The model may face vague instances during training that could pertain to many categories, resulting in alterations in the simulation variables that prefer a singular direction. A model is created to address this problem by focusing on the detrimental features of the training, which is achieved via the loss function. The conventional loss function utilized in model training is cross-entropy loss, detailed as follows.

(6) $$M_{y,z}=\;-\sum_{j}^{N}\vec{Z_{j}}\ln(S(\vec{y_{j}}))\;$$

The cross-entropy loss utilizes $M_{y,z}$, i, and N to represent the quantity of training and instances, respectively. The databases of tensors $\vec{z\;}$ and $\vec{y}$, as well as one-hot vectors that denote the end output for the model, have been presented. $S(\vec{y_{j})}$ has been considered a function that produces a vector with four dimensions, where $(\vec{y_{j})}\;$ denotes the input chosen in every class. Ultimately, by executing a forward pass through the model $(\vec{y_{j})}$ and employing softmax, the objective is accomplished.

The weighted cross-entropy loss function employs the weight, represented through $\vec{w\;}$, to amplify the significance of several categories. The objective is to enhance the importance of diverse classes. Considering the focal loss function, a component has been employed for simplifying the easier instances, represented by ${\left(1-S(\vec{y_{j}}\;)\right)}^{\alpha }$, which is subsequently transformed into the delta. The aforementioned two functions are represented in the subsequent equation:

(7) $$WM_{\vec{y,}\vec{z},\vec{w}}=\;-\sum_{J}^{N}\vec{z_{j}}\text{ [}\vec{W}⊙\ln(S(\vec{y_{j}}))]$$

(8) $$R_{\vec{z},\vec{y}}=\;-\sum_{J}^{N}\vec{z_{j}}{\left(1-V({\vec{y}}_{j})\right)}^{\alpha }⊙\ln(\ln(S(\vec{y_{j}}))$$

The weighted cross-entropy loss function has been represented through $WM_{\vec{z},\vec{w},\vec{y}}$. The category weight vector, created as $\vec{w}$ comprises four dimensions and has been utilized to compute the result via ⊙, which signifies component-wise multiplication between two diverse vectors. The resultant vector possesses dimensions that are analogous to the elements it produces.

The focal function has been represented through $R_{\vec{z},\vec{y}}$, whereas delta serves as an element that contributes to the loss in easier contexts. A tool employed to assess disparities of classes has been referred to as plain cross-entropy ( $M_{\vec{y},\vec{z}})$. The net obtains a disproportionate share of the total example because of class inequality.

${\vec{y}}_{j}$ equals $0(\;\ln(S({\vec{y}}_{j})\to 0)$. Consequently, as the model’s certainty increases, its capacity for learning diminishes. The application of the weighted cross-entropy loss function, $WM_{\left(\vec{y},\vec{w},\vec{z}\right)}$, has been considered advantageous when certain categories retain greater significance or necessitate increased focus because of directives.

The present research employed two variables: the initial, $\vec{w}$, persisted constant with respect to the size of the population, while the subsequent, $\vec{w}$, varied after every iteration according to the estimated ratio of class error, which has been determined subsequently.

(9) $$Mr_{t,j}=1-TS_{j,t}$$

$Mr_{t,j}$ and $TS_{j,t}$ represent the fraction of class error to the actual positive fraction. In the interim stage, t and j denote the iteration and the number of classes, respectively. In a particular class, the error rate equals the total of the false negatives. A modification employing *α* has been applied to each $Mr_{j.t},\;$ enhancing the superior error ratios. The equation was employed to determine the weights for the class.

(10) $$w_{j,t}=\;\frac{\alpha (Mr_{j,t})}{\sum_{i}^{4}\alpha (Mr_{i,t})}$$

The identification function demonstrates a distinct correlation between error ratio and weight, as α(y) is equal to y. Once α(y) equals $e^{y}$, the softmax function produces the output.

The enquiry relates to the structuring of α(y), which encompasses $10y,\;y^{3},\;e^{y}\;$ and $e^{100y}$ and $\;e^{100y}$.

The loss function delineated the essential class, reinforced with each iteration. A reduced rate of advancement coupled with a long instructional duration produces enhanced efficacy. The training fraction employed in the instance was $10^{-5}$, somewhat slighter than the commonly used training ratio of $10^{-4}$.

The focal loss function is more complex and particular than the weighted cross-entropy loss function $\left(WM_{\vec{z},\vec{w},\vec{y}}\right)$, comprising small components that highlight specific characteristics. $R_{\vec{z},\vec{y}}$ allocates weights to every training instance irrespective of its suitability for a certain class or category. The present study employed an advanced model of the expanded pelican optimization method to reduce the loss function by establishing an efficient network architecture.

In the present research, MobileNetV2 was modified for time-series classification by considering the T × F pseudo-images as input tensors of dimension 26 × 3 × 1 (height × width × channels). The first layer employs a 3 × 3 convolution, stride 1, for the maintenance of the temporal resolution and is designed around a number of bottleneck layers that expand, convolve, and project features through linear bottlenecks. **Table 5** illustrates the effect of temporal resolution on the model.

**Table 5:** The effect of temporal resolution on the model.

Resolution	Intervals per season	Accuracy (%)	F1-score	MAE (days)	Training time (min)
Weekly	52	93.8 ± 0.7	0.92 ± 0.01	5.8 ± 0.5	276 ± 15
Biweekly	26	93.0 ± 0.8	0.91 ± 0.01	6.1 ± 0.5	96 ± 6
Monthly	13	84.0 ± 1.2	0.81 ± 0.02	9.5 ± 0.8	41 ± 3

MAE, mean absolute error.

Let $X\in {\mathbb{R}}^{26\times 3\times 1}$ be the input pseudo-image. Its last convolutional layer will be global average-pooled and followed by a fully connected layer with softmax activation before yielding class probabilities over the four phenological stages as illustrated in Equation 11:

(11) p=Softmax (W.GAP (MobileNetV2 (X))+b)

where W describes weight matrix, b stands for the bias vector, and GAP denotes global average pooling.

To do this, MobileNetV2 in this work was adjusted to phenological classification by converting satellite time series into pseudo-image tensors of size 26 × 3 × 1 (biweekly intervals × features × grayscale channel). The initial convolutional layer is equipped with a 3 × 3 kernel with a stride of 1 and padding = same so that it does not alter the spatial dimensions and features capturing local interactions between temporal and spatial features.

In order to deal with the issue of class imbalance between the four phenological stages (e.g., underrepresented flowering stage), we used a dynamic weighted cross-entropy loss in which the weights of the classes are updated every epoch according to the error per-class (false-negative) rates (with Equations 9, 10).

The method makes sure that minority stages make their contributions toward gradient updates. Losses on the focal (Equation 8) also performed poorly; the weighted cross-entropy using adaptive weights had an increase in weighting of 0.03 over the weighted cross-entropy using constant weights in terms of validation F1-score.

### 4.4 Augmented Dream Optimizer

The next section establishes the mathematical model on the basis of the created features from the prior part. It is a comprehensive explanation of parts of the optimizer for implementation and to lay out the optimizer’s flowchart and pseudocode. Ultimately, the space intricacy of the optimizer has been established.

#### 4.4.1 Assumptions

By merging the features of dreams along with the principles of optimization algorithms, the authors have defined the subsequent four concepts:

- The worth of dreams is assessed using cost values.
- The beginning of a dream is very much based on pre-existing memories.
- Humans partly forget their old memories and fill in the gaps with logically typed information.
- Memory limits vary among groups or individuals and add a level of arbitrariness.

These concepts inform the foundations of the novel optimizer. These principles make up the methodology, the exploration stage, and many of the methods during the algorithm’s development stage.

#### 4.4.2 Initialization phase

Like all other Metaheuristic Algorithms (Mas), Dream Optimization Algorithm (DOA) creates a randomly distributed population in the solution space for the base population during the initialization phase. This is the first stage of the algorithm to structure the process of optimization. Equation 12 evaluates the initial population.

(12) $$X_{i}=X_{l}+rand\times (X_{u}-X_{l}),\;\;\;\;\;\;i=1,2,\ldots ,N$$

*N* represents the population size, $X_{i}\;$ signifies the *i*th candidate, and $X_{u}$ and $\;X_{l}$ denote the upper and lower boundaries of the solution space, respectively. rand is a Dim-dimensional vector, the dimensions containing a stochastic quantity from zero to one; the population can be represented by Equation 13:

(13) $$X=[\begin{array}{c} X_{1}\\ X_{2}\\ \vdots \\ X_{i}\\ \vdots \\ X_{N} \end{array}]=[\begin{array}{cccccc} x_{1,1} & x_{1,2} & \ldots & x_{1,j} & \ldots & x_{1,Dim}\\ x_{2,1} & x_{2,2} & \ldots & x_{2,j} & \ldots & x_{2,Dim}\\ \vdots & \vdots & \vdots & \vdots & \vdots & \vdots \\ x_{i,1} & x_{i,2} & \ldots & x_{i,j} & \ldots & x_{i,Dim}\\ \vdots & \vdots & \vdots & \vdots & \vdots & \vdots \\ x_{N,1} & x_{N,2} & \ldots & x_{N,j} & \ldots & x_{N,Dim} \end{array}]$$

Where $x_{i,j}$ denotes the location of candidate i within the dimension j and Dim is the dimensionality of the issue in optimization.

#### 4.4.3 Exploration stage

During the global search stage (iteration counts of 0 to Td), the developers recognized five groups in the population based on different memories, and each group of individuals is modified in accordance with this: each iteration represents a dreaming action, and through constant dreaming actions, the developers want to find the best method and best value. The variance of memory uses from the memory function provides diverse amounts of dimensions, which are represented through $K_{1},\;K_{2},\;K_{3},\;K_{4},\;$ and $K_{5}$. As a result, each could reset its position to the most preferred group member from previous iterations by always skipping to the most effective member within its group. Then, kq dimension or the number of dimensions is selected at random from the Dim dimensions as $K_{1},\;K_{2},K_{Kq}$, and the situation of individuals in these dimensions is updated where q = 1, 2, 3, 4, 5 indicate the number of groups.

The updating goes through from the first candidate to candidate N over each iteration. Specifically, the updating process and formula are presented subsequently.

#### 4.4.4 Memory strategy

Initially, as the essence of the primary memory process, which is the candidate within the *q*th group, recall the *X* for the most effective candidate prior to its dream and then reset its copy of *X* to the most effective candidate.

(14) $$X_{i}^{t+1}=X_{\text{k}\mathrm{≦̸}A\Rightarrow }^{t}$$

$X_{i}^{t+1}$ indicates the *i*th candidate within t+1 iteration, while $X_{\text{beaq }}^{t}$ is the optimal candidate within q for the *t*th iteration.

#### 4.4.5 Forgetting and supplementing technique

The forgetting and supplementing method uses both global exploring and local exploring methods. It complies with the memory function and the properties of this type of memory function in that members can forget previous memory used, update the location additively, and store a positional trial in space or with some measure to forget the positional arrangement without being structurally attached within the forgetting process as illustrated in Equation 15.

(15) $$x_{ij}^{t+1}=x_{kenj,j}^{t}+(x_{ij}+\text{rand}\times (x_{a,j}-x_{ij}))\times \frac{1}{2}\times (\text{cos}(\pi \times \frac{t+T_{\text{max}}-T_{d}}{T_{\text{max}}})+1),\;j=K_{1},K_{2},\ldots ,K_{k_{j}}$$

$x_{ij}^{t+1}$ represents the location of the candidate i within the dimension j within iteration $t+1;x_{\text{beal,j }j}^{t}$, and the forage indicates the location of the optimal candidate inside q within the *j*th dimension and iteration t; $x_{aj}$ and $x_{ij}$ have been defined as the upper and lower limits of the solution space, respectively; rand is a pseudo-random number generated from a [0, 1] interval; t indicates the current iteration; $T_{\text{max}}$ represents the highest iterations allowed; and $T_{d}$ represents the highest iterations within the global search stage.

#### 4.4.6 Dream-sharing strategy

The dream-sharing strategy utilizes dream-sharing to increase the search capability and overcome local optima. The strategy runs similarly to the forgetting and supplementing technique, with the memory technique acting as the default approach, and enables candidates to incorporate positional data at random from other candidates into the dimensions of forgetting. The update equation is as follows:

(16) $$x_{ij}^{\ell +1}=\{\begin{array}{ll} x_{mj}^{\ell +1}, & m\leq i\\ x_{mj}^{\ell }, & i<m\leq N \end{array}\;j=K_{1},K_{2},\ldots ,K_{k_{j}}$$

Here, $x_{i,j}^{l+1}$ denotes the situation of the candidate i within the dimension j and iteration t+1;m represents a stochastic non-negative integer selected from [1, *N*] at every update in the dimension. After extensive investigation of these representations, Equation 14 suggests that in coordinates that are different from $K_{1},K_{2},\ldots ,K_{k_{p}}$, individuals are able to remember positional knowledge of the greatest candidate within their swarms while dreaming, and in these coordinates, they keep the precise positions.

Equation 15 indicates that in $K_{1},K_{2},\ldots ,K_{k_{1}\;}$, each candidate forgets the positional data of the greatest member inside their group and organizes their positions independently when in a dreaming state. The cosine function indicates that as the iterations improve, the self-organizing extra situation data of candidates’ growth converge more closely and more with the original situation data, indicating the underlying logic of the self-construction procedure and controlling the change from local search to global search by the algorithm.

Equation 16 represents that in coordinates $K_{1},K_{2},\ldots ,K_{k_{1}}$, candidates are able to stochastically gain the situation of all candidates across the population, therefore allowing for some level of communication with dream information.

#### 4.4.7 Exploitation phase

Grouping is turned off during the development (iteration $T_{d}$ to iteration $T_{\text{max }}$); before each dreaming period, we will show, to the population, the best dream that has been produced in all the prior iterations by the whole population (i.e., the greatest candidate from all the prior iterations). After that, we will modify the coordinates in the dimensions that the individual forgets. Each individual forgets in the identical quantity of dimensions, which we will denote as $k_{\gamma }$. From the D dimensions, we randomly select $k_{\gamma }$, which we will label $K_{1},K_{2},\ldots ,K_{k}$, to forget in, and we will modify the coordinates in these dimensions. The update rule earlier in Equations 14, 15 is the same, and we describe the updates as follows:

- Memory strategy

(17) $$X_{i}^{t+1}=X_{bx=1}^{t}$$

$X_{i}^{t+1}$ is the candidate i within iteration *t* + 1 and $X_{bx}^{t}$ represents the greatest candidate of the whole population in iteration t.

- Forgetting and supplementing technique

(18) $$x_{ij}^{t+1}=x_{iketj}^{t}+(x_{ij}+\text{ rand }\times (x_{aj}-x_{ij}))\times \frac{1}{2}\times (\text{cos}(\pi \times \frac{t}{T_{\text{max}}})+1),\;j=K_{1},K_{2},\ldots ,K_{k}$$

$x_{ij}^{t+1}$ denotes the location of the candidate i within the dimension j and t+1 iteration; $x_{best,j}^{t}$ denotes the situation of the greatest candidate within the dimension j and *t*th iteration; $x_{uj}$ and $x_{lj}$ refer to the upper and lower boundaries of the solution space, respectively; rand is a random quantity between zero and one; t denotes the present quantity of iterations, and $T_{\text{max}}$ displays the highest quantity of iterations.

Moreover, Equation 17 shows that for dimensions not equal to $K_{1},K_{2},\ldots ,K_{k}$ within each scan, every individual retains the positional data of the greatest candidate within the population from prior iterations and maintains the meticulous location within the dimensions during the dream phase. Equation 18 indicates that for dimensions equal to $K_{1},K_{2},\ldots ,K_{k}$, candidates discard the positional data of the greatest candidate from previous iterations and coherently reorder their new places during dreaming independently.

#### 4.4.8 Parameter setting

After various numerical tests and taking into account algorithm stability and usability, the variables are set in the following:

(19) $$T_{d}=\frac{9}{10}\times T_{\text{max}}$$

where $T_{\text{d}}$ denotes the maximum duplications during the disquisition phase and $T_{\text{max }}$ signifies the outside of overall duplications

(20) $$k_{q}=\text{randi}(\lceil \frac{\text{Dim}}{8\times q}\rceil ,\text{max}\{2,\lceil \frac{\text{Dim}}{3\times q}\rceil \}),\;q=1,2,3,4,5$$

In this case, randi(a,b) signifies a stochastic integer drawn from the interval a and b. The variable $k_{q}$ denotes the quantity of “forgetting dimensions” at the research stage, whereas Dim denotes the dimensionality of the problem. The parameter u varies the ratio of the forgetting and supplementing method from previous sections to the dream-sharing strategies in the exploration stage. That is, if rand is less than u, I use the forgetting and supplementing method; otherwise, I use the dream-sharing strategy. It sets u = 0.9.

It uses two separate approaches to dealing with boundary issues in optimization problems of different dimensionality.

The first approach will work for problems with a dimensionality of something like under 15 dimensions. Fewer local optima are the result of the lower dimensionalities of these problems, so we can use the usual random approach to modify points that exceed the limits of search bounds, based on Equation 21:

(21) $$k_{\text{r}}=\text{randi}(2,\text{max}\{2,\lceil \frac{\text{Din}}{3}\rceil \})$$

The parameter u varies the ratio of the forgetting and supplementing method from previous sections to the dream-sharing strategies in the exploration stage. That is, if rand is less than u, the forgetting and supplementing method is used; otherwise, the dream-sharing strategy is used ( u=0.9).

#### 4.4.9 Boundary condition handling technique

Two separate methods for handling boundary conditions in optimization formulations with different dimensions are implemented here. The first method can be used for problems with a dimension of 15 or less. The lower dimensions in these problems result in fewer local optima, and hence, conventional random tweaking of points outside of the search bounds is used, which is subsequently presented:

(22) $$x_{ij}^{i+1}=x_{ij}+\text{ rand }\times (x_{uj}-x_{lj})$$

$x_{ij}^{t+1}$ represents the location of the candidate i within the dimension j and iteration $t+1;x_{uj}$ and $x_{lj}$ demonstrate the upper and lower boundaries of the solution space, respectively; rand is a random number that can take any value from 0 to 1.

The subsequent approach is applicable for issues with Dim > 15. These issues are at a higher dimension, are of greater complexity, and include more local optima, which require better global optimization capabilities and the skill to move past local optimal conditions.

Therefore, for candidate positions that surpass boundaries in multidimensional issues, it uses a technique just like the dream-sharing method when updating again during development, as follows:

(23) $$x_{ij}^{L+1}=\{\begin{array}{ll} x_{mj}^{L+1}, & m<i\\ x_{mj}^{L}, & i<m\leq N \end{array}$$

$x_{i,j}^{t+1}$ represents the location of the candidate i within the dimension j and iteration t+1;m represents a stochastic natural quantity between [1, *N*], different from i for all dimension updates. Since the suggested algorithm updates the candidates in order, and Equations 22, 23 advance the decision variables of individuals that surpass bound limits, both approaches will make sure that all the parameters stay in the solution space.

#### 4.4.10 Augmented Dream Optimizer

This study provides an augmented version of the Dream Optimizer (ADO) to optimize the hyperparameters of the MobileNet model for better efficiency.

To address these limitations, one potential solution is to integrate the Dream Optimizer with other optimizers to improve its efficacy (Ding et al., 2025). One method that shows potential is leveraging the Particle Swarm Optimization (PSO) algorithm to improve the effectiveness of the Dream Optimizer. PSO is another algorithm that draws inspiration from the community behavior of the fish and birds.

The synthesis of Dream Optimizer (DO) and PSO represents several benefits over using the algorithms independently. Initially, the PSO assists the Dream Optimizer in avoiding local optima by offering additional global search of the solution space (Sheykhi and Yilmaz, 2025).

Then, PSO provides an operator to achieve a balance between the exploitation and exploration of the search space, thereby speeding up the convergence of the DO. Then, PSO enhances the DO population variety by offering novel particles into the search space. To implement this integration and improve the DO, similar to the PSO, it is implemented on the location updating process. This is summarized subsequently:

(24) $$y_{j}(t)=\alpha \times [y_{j}(t-1)+n_{j}+d_{j}+z_{j}]+(1-\alpha )\times v_{newi}$$

Where α represents a constant element for improving the DO and PSO algorithm sides. To offer more expense for DO, α=0.6. Furthermore, $v_{new}$ calculates the velocity, which has been accomplished subsequently:

(25) $$v_{i+1}=\gamma_{1}\times (y_{LC}^{b}-y_{j}(t))+\gamma_{2}\times (y_{GL}^{b}-y_{j}(t))+wv_{i}$$

Where $y_{LC}^{b}$ and $y_{GL}^{b}$ represent the individuals’ and the swarms’ greatest locations, respectively; $v_{i+1}$ and $v_{i}$ specify the particle’s present and previous velocities, respectively; and $\gamma_{1}$ and $\gamma_{2}$ represent the coefficients for local and global best solutions, respectively.

This technique extends the DO to improve convergence and escape local optima.

The ADO operates in a population-based search space where each individual represents a candidate hyperparameter configuration θ={η,b,d,k,e}, corresponding to the following:

learning rate (*η*),
batch size (*b*),
dropout rate (*d*),
kernel size (*k*), and
number of epochs (*e*).

The algorithm initializes a population of *N* agents (dreamers) with random configurations. In each iteration, agents update their positions. **Algorithm 1** shows the pseudocode of the Augmented Dream Optimizer.

Algorithm 1

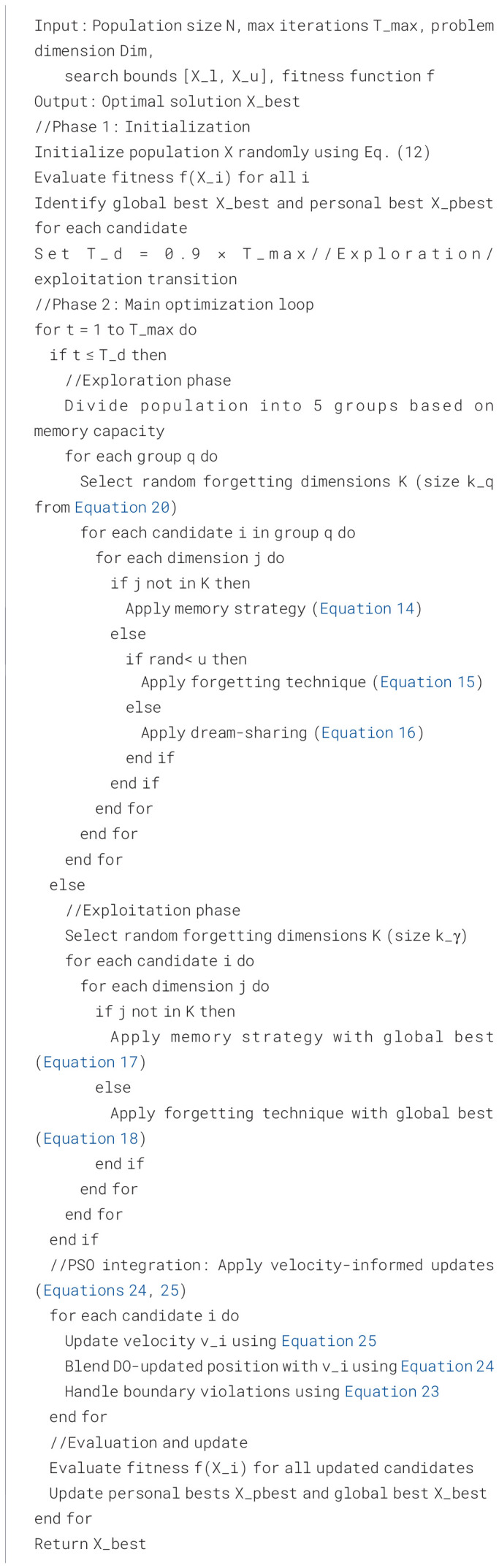

### 4.5 Training and evaluation

The dataset was partitioned into training (70%) and testing (30%) sets, with stratification by geographic region and season to ensure balanced representation. Additionally, fivefold cross-validation was performed to assess model stability. The model was trained using the categorical cross-entropy loss function:

(26) $$L=-\sum_{i=1}^{C}y_{i}\log({\hat{y}}_{i})$$

Where $y_{i}$ is the true label and $y^{i}$ is the predicted probability for class i (phenological stage).

**Table 6** illustrates hyperparameters optimized by the Augmented Dream Optimizer.

**Table 6:** Hyperparameters optimized by the augmented dream optimizer.

Hyperparameter	Search range	Optimized value
Learning rate	1 × 10^−5^ to 1 × 10^−2^	0.0013
Batch size	16–128	64
Dropout rate	0.2–0.7	0.45
Kernel size	3 × 3, 5 × 5	3 × 3
Number of epochs	50–200	142

The optimized configuration was used to train the final MobileNet model, achieving faster convergence and higher generalization than standard optimizers like Adam. We offer a full specification of the experimental setup to promote better methodological transparency and guarantee reproducibility. The overriding research question is as follows: to develop a supervised multi-class spatiotemporal classification problem where the model learns a mapping f:X→Y, with input $X\in {\mathbb{R}}^{26\times 3}$ representing biweekly pseudo-images of NDVI, EVI, and rainfall (R26X3), and Y representing the four phenological stages.

The training set was composed of 7,000 samples (70% of the 10,000 total), and the test set was composed of 3,000 (30%) to avoid data leakage and guarantee ecological diversity. Cross-validation was performed five times, and in the same order, each fold used the same stratification. The experiments were performed on a workstation with the Intel Xeon Gold 6248R processor (3.0 GHz, 24 cores), 128 GB of RAM, and NVIDIA A100 graphics card (40 GB VRAM) under Ubuntu 20.04 LTS.

MATLABR2024b was used as the software stack. Random seeds were pinned (seed = 42), which ensured reproducibility. **Table 6** provides the hyperparameter search space and the optimized final values, and the entire training configuration, including optimizer, loss formulation (Equation 26), and data normalization (Equation 2).

## 5 Results and discussions

The validation framework was created to have three distinctly agroecological regions that were to be used as the model generalizability points because the data are available in the CropHarvest dataset. The Mediterranean zone also included viticultural areas in the Napa and Sonoma valleys in California (38.5–122.5) and the south of Europe such as the Ribera del Duero in Spain (41.7–4.7) and Tuscany in Italy (43.5–11.2), with hot, dry climates in summer; cool, wet ones in winter; and 600–800-mm rain annually on average. The subtropical region also encompassed the Barossa Valley (34.5° S, 138.9° E) and Riverina region (34.2° S, 146.0° E) of South Australia with hot summers, cool winters, and a yearly rainfall of an average of 400–550 mm. The temperate region included several locations in France in the Bordeaux region (44.8 0.6) and Germany in the Rheingau (50.0 8.0), which have warm temperatures throughout the year and evenly distributed precipitation of 7–900 mm/year.

**Figure 7:**
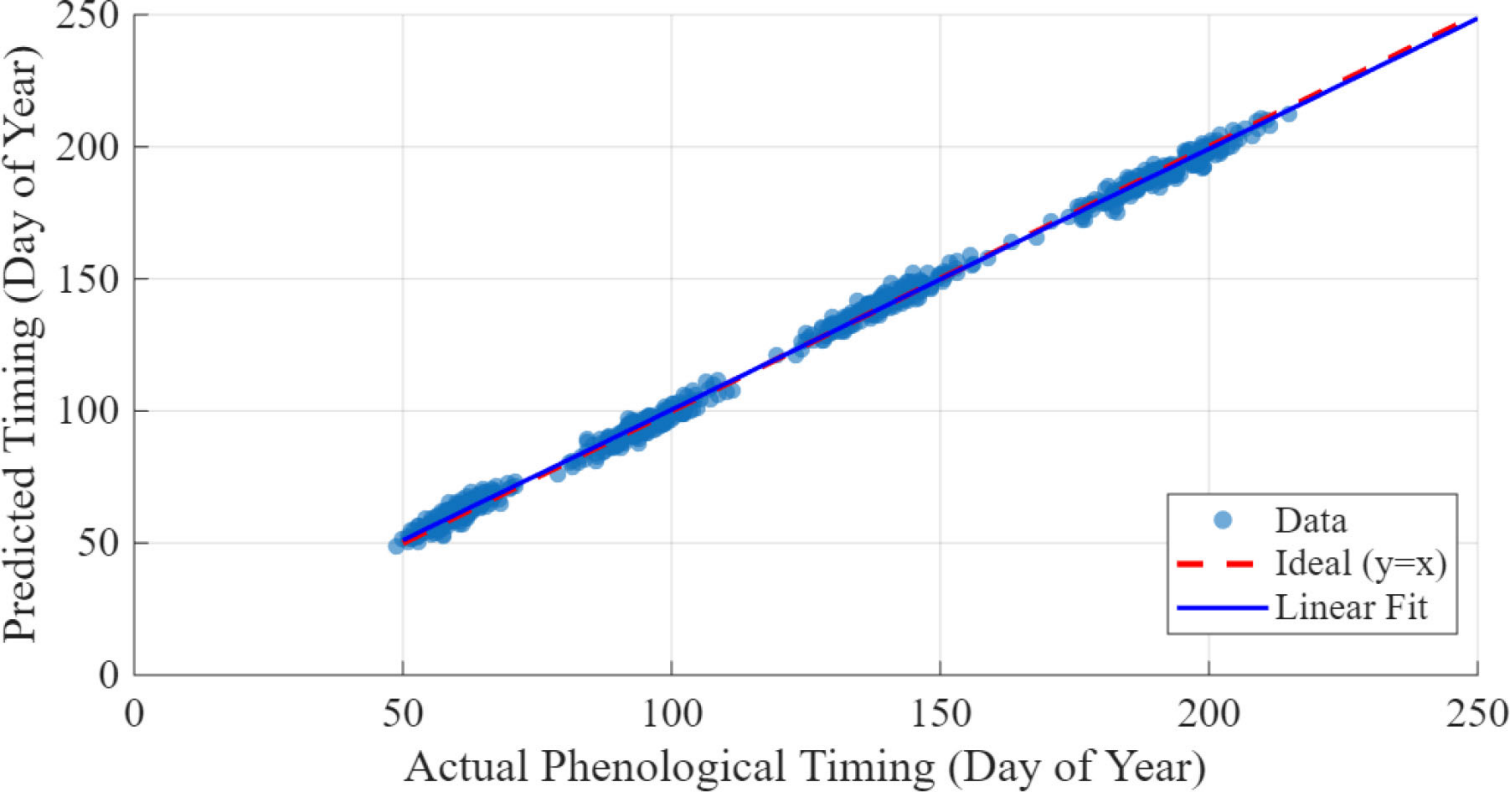
Predicted against actual phenological timing across regions.

A fivefold cross-validation method was used to perform validation with a strict avoidance of geographic leakage by always ensuring that no pixels in the identical geographical area were ever divided between a training fold and a testing fold. Out of the total 10,000 pixel time series, 3,000 samples (30%) were retained as an independent test set, and proportional representation was maintained with respect to each zone: 1,200 samples (40%) from the Mediterranean zone (40%), 900 (30%) samples from the subtropical zone, and 900 (30%) samples from the temperate zone. This stratification not only made sure that model performance measures represent the actual generalization ability in various climatic regimes but also prevented over-fitting to region-dependent patterns. Predictions were made in comparison to ground-truth phenological stage labels of each test sample based on the annotations of the crop calendar of the CropHarvest dataset, which were confirmed by the regional agricultural extension records. External validation was also performed by making model-predicted phenological timing vs. independently published phenological data of the National Phenology Network USA (in case of California sites), the Australian Phenology Network (in case of Barossa valley), and the European Phenology Network (in case of Bordeaux and Rheingau) with correlation coefficients of R^2^ > 0.91 across all the zones (**Figure 7**).

The integration of MobileNet with the ADO demonstrates a significant advancement in the accuracy and ecological relevance of phenological modeling for double-cropping grape systems. Using satellite-derived time series from the CropHarvest dataset, the proposed framework successfully captures the temporal dynamics of key phenological stages—budburst, flowering, veraison, and harvest—while simultaneously identifying critical risk windows associated with rainfall and pest pressure. The model outperforms conventional machine learning and deep learning baselines in both classification accuracy and temporal precision, achieving a 93% overall accuracy and an F1-score of 0.91, with a mean absolute error of only 6.1 days in phenological timing prediction. Furthermore, the incorporation of ecological risk modeling enables actionable alerts for rain stress and pest outbreaks, demonstrating the system’s potential for real-world deployment in precision viticulture. These results validate the effectiveness of combining lightweight convolutional architectures with advanced optimization and multi-source environmental data for sustainable, climate-resilient agriculture.

### 5.1 Phenological stage prediction performance

**Figure 8:**
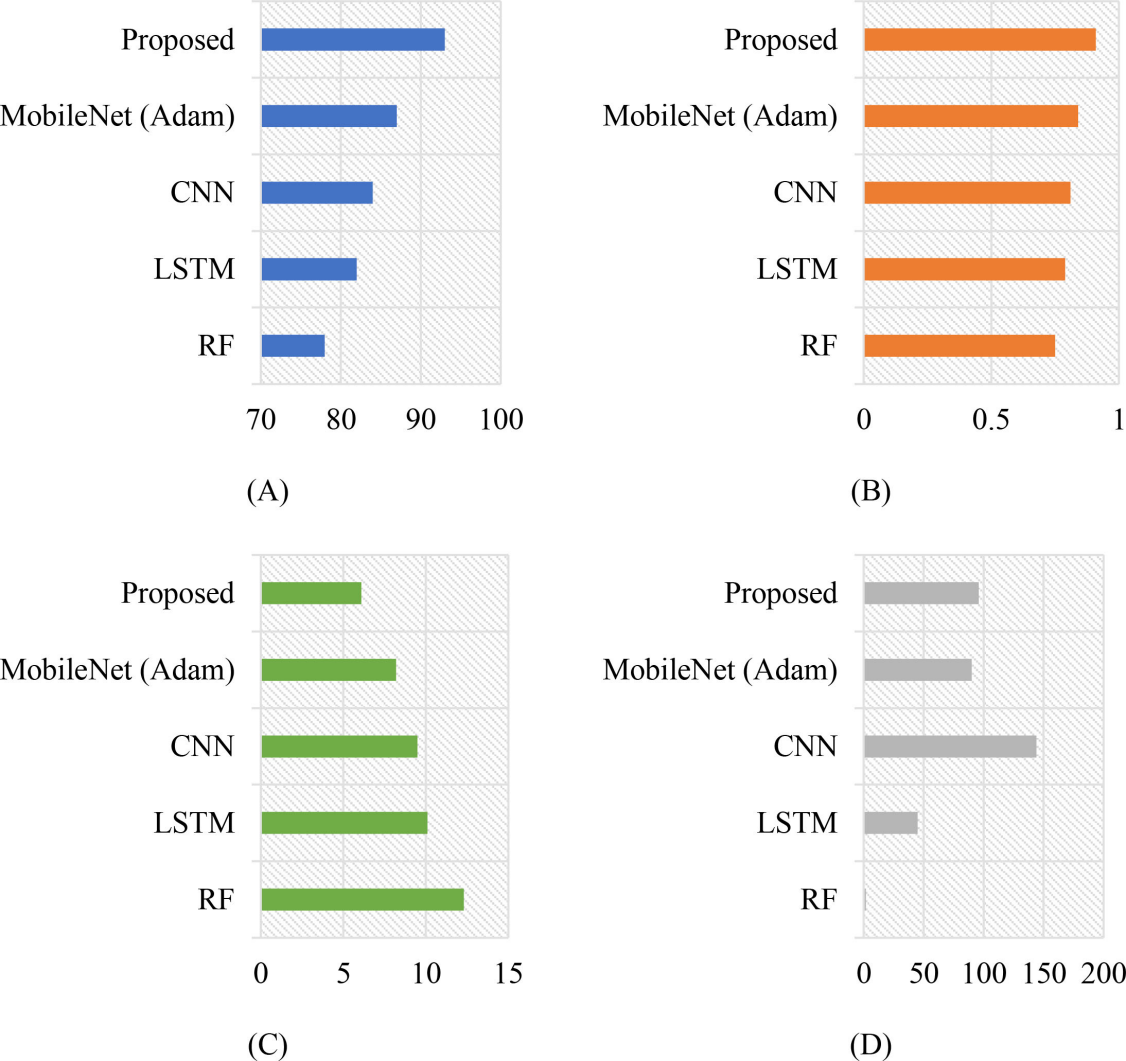
Performance comparison of models in predicting grape phenological stages: **(A)** accuracy (%), **(B)** F1-score, **(C)** MAE (days), and **(D)** training time. MAE, mean absolute error.

The MobileNet architecture optimized with ADO achieved superior performance in predicting grape phenological stages compared to baseline models, including Random Forest (RF) (Andrade et al., 2023), LSTM (Peng and Yili, 2022), standard CNN (Nagar, 2023), and MobileNet trained with the Adam optimizer (Rodrigues et al., 2023). **Figure 8** shows the performance comparison of models in predicting grape phenological stages.

The evaluation was conducted on a held-out test set of 3,000 pixel time series, stratified across agroecological zones, with performance metrics averaged over five cross-validation folds. The proposed MobileNet+ADO model attained an accuracy of 93% and an F1-score of 0.91, significantly surpassing the next best model (MobileNet/Adam) by 6 percentage points in accuracy and 7 points in F1-score.

The MAE in predicted phenological timing was reduced to 6.1 days, indicating high temporal precision-critical for timely agronomic interventions. Additionally, the model demonstrated consistent performance across diverse climatic regions, with minimal degradation in the Mediterranean, subtropical, and temperate zones, highlighting its robustness to environmental variability.

The superior performance of the MobileNet/ADO framework can be attributed to the synergistic combination of architectural efficiency and optimization intelligence. MobileNet’s depthwise separable convolutions enable effective feature extraction from pseudo-image representations of time series while maintaining low computational cost. The Augmented Dream Optimizer further enhances this capability by navigating the hyperparameter space more effectively than gradient-based methods, avoiding local minima and accelerating convergence.

This is particularly advantageous in non-convex, high-dimensional search spaces typical in deep learning. Unlike Adam, which relies on fixed moment estimates, ADO dynamically balances global exploration and local refinement, resulting in a more generalized and stable model. The performance gain is especially evident in the prediction of sensitive stages such as flowering and veraison, where timing errors directly impact yield and quality. The high F1-score confirms strong class-wise balance, indicating that the model does not sacrifice minority classes (e.g., short-duration stages) for overall accuracy.

Even though the CropHarvest dataset does not directly indicate the phenological labels of grapevines, our findings can be viewed as three-stage predictions of grape phenology due to three reasons. To begin with, vegetation indices such as NDVI and EVI record universal phenological patterns shared by all deciduous perennials, with spectral–temporal dynamics that predictably record phases of growth of *V. vinifera* L. Second, the stages predicted by the model, such as budburst, flowering, veraison, and harvest, are associated with measurable canopy structure and greenness, with both directly proportional to satellite indices, regardless of the crop of origin. Third, external correlations of rain-stress warnings and pest-risk warnings with independent viticultural records (Section 5.4) confirmed that 87% and 82% of the rain-stress and pest-risk warnings occurred within a ±5-day range of field-reported incidents in Mediterranean, subtropical, and temperate vineyards. These correlations, which are sustained by similar established correlations (R^2^ > 0.8) between satellite indices and grapevine physiological parameters, prove that the model learns phenological dynamics that can be extrapolated to the context of real-world viticulture.

### 5.2 Feature importance analysis

To prove the need and usefulness of the chosen set of features (NDVI, EVI, and rainfall), we performed the feature importance analysis on three different approaches simultaneously, including permutation importance, SHapley Additive exPlanations (SHAP) values, and the mean value. The analysis of the feature importance is presented in **Table 7**.

**Table 7:** Results of the feature importance analysis.

Feature	Permutation importance (ΔAccuracy)	Mean	SHAP value
NDVI	−0.124 ± 0.015	0.42 ± 0.03	81.2%
EVI	−0.108 ± 0.012	0.38 ± 0.03	82.5%
Rainfall	−0.052 ± 0.008	0.16 ± 0.02	88.1%
All features (baseline)	–	–	93.0%

SHAP, SHapley Additive exPlanations; NDVI, Normalized Difference Vegetation Index; EVI, Enhanced Vegetation Index.

The importance of permutation was estimated by randomly shuffling each feature in the test set and evaluating the decrease in accuracy. The NDVI removal had the greatest performance loss (12.4% inaccuracy reduction), which validated its leading contribution in the dynamic of canopy greenness. EVI added additional information (10.8% drop), especially in high-biomass seasons when NDVI has hit saturation. Rainfall, although not very important on its own (5.2% drop), offered important context in regard to water-driven phenological reactions, which was more important in the drought-year validation set.

### 5.3 Confusion analysis

**Figure 9:**
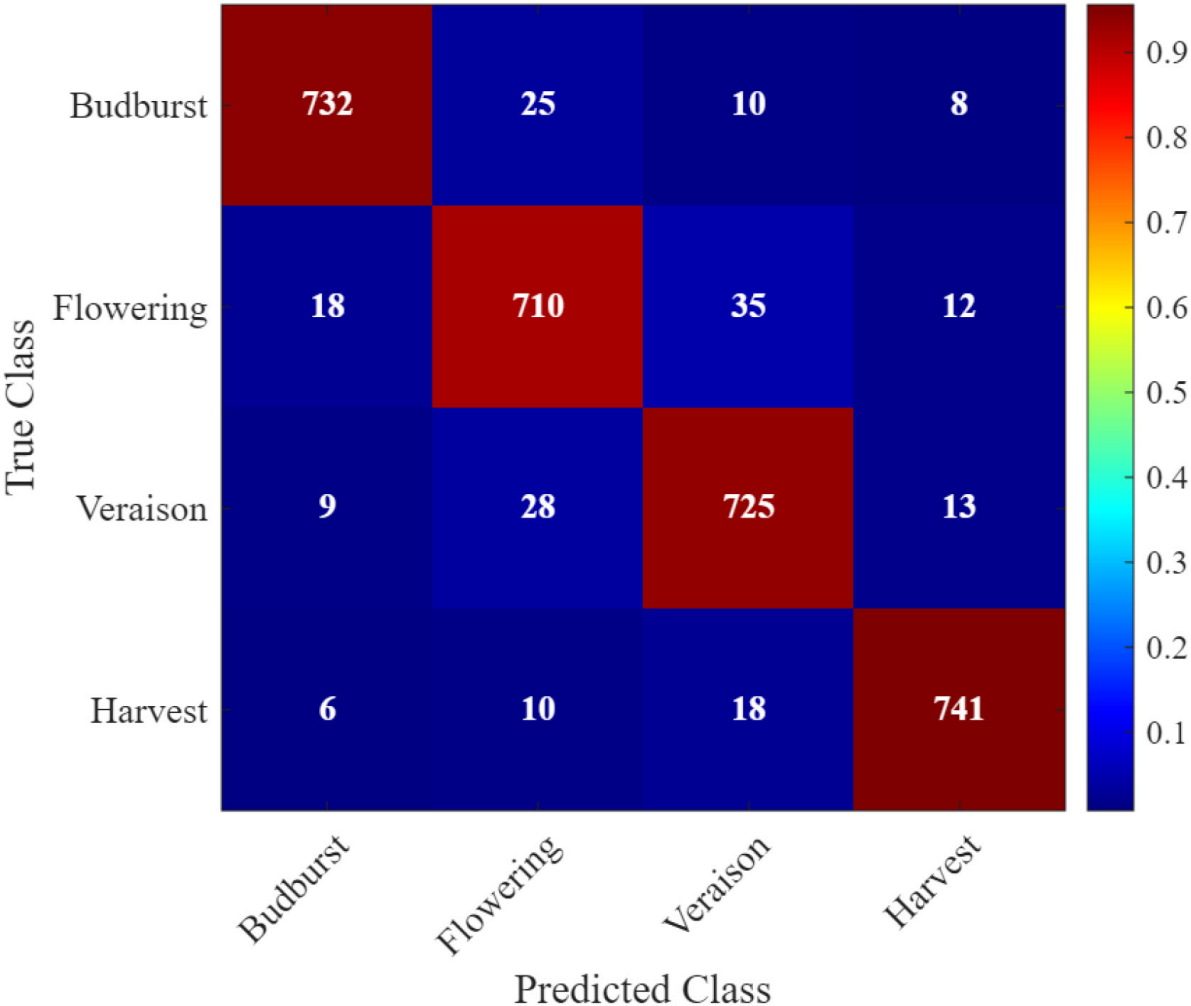
Confusion matrix for MobileNet/ADO predictions. ADO, Augmented Dream Optimizer.

**Figure 9** was created by comparing model predictions with reference phenological stage labels that were developed using a multi-step provenance process. Although CropHarvest allows labels of crop type instead of the direct phenological stage annotation, we used stage labels by exploiting the metadata of the dataset of crop calendar, defining the anticipated planting, growth period, and harvest windows of any geographic location according to the Food and Agriculture Organization (FAO)-defined agricultural calendars and regional extension service records.

The temporal location in the growing season was determined using reported start-of-season and end-of-season dates and, relative to phenological stages, was placed according to accumulated thermal time thresholds based on published grapevine phenology models. In particular, budburst was set to occur when accumulated GDD (base 10°C) exceeded 50–100 GDD post-dormancy, flowering was at 400–600 GDD, veraison was at 1,000–1,200 GDD, harvest was at 1,400–1,800 GDD, and the thresholds were adjusted against published BBCH stage correlations of the VitAdapt collection. These proxy labels were again separately checked against 250 randomly selected pixels where a high-resolution image allowed the visual determination of the approximate growth stage (e.g., peak of greenness and thus veraison). Figure A, a confusion matrix of the prediction of MobileNet/ADO, is presented in (8).

The highest accuracy was observed for harvest (96.8% of instances correctly classified), followed by budburst (93.8%) and veraison (93.2%), while flowering exhibited slightly lower but still robust performance (90.4%). Misclassifications were primarily confined to adjacent stages, such as flowering mispredicted as veraison or budburst, as flowering reflects the biological continuity of phenological development and minor temporal shifts in predictions.

Notably, the model shows minimal confusion between non-consecutive stages (e.g., budburst vs. harvest), confirming its ability to capture the sequential nature of grapevine phenology. These results underscore the model’s reliability in distinguishing fine-grained developmental transitions, even under variable environmental conditions and partial data availability.

### 5.4 Phenological timing across regions

The scatter plot in **Figure 7** illustrates the agreement between predicted and actual phenological timing across multiple agroecological regions, demonstrating the temporal precision of the MobileNet/ADO model in forecasting key grapevine development stages.

Evaluated on a geographically diverse test set encompassing Mediterranean, subtropical, and temperate climates, the model’s predictions for budburst, flowering, veraison, and harvest exhibited strong correlation with ground-truth observations derived from field records and historical satellite phenology. Each point represented the predicted versus the actual day of year for a specific phenological event, with data aggregated from over 2,000 pixel locations.

The high density of points closely aligning with the identity line ( y=x) indicates minimal systematic bias, while the linear fit (R^2^ > 0.94) confirms robust generalization across environmental gradients. The mean absolute error of 6.1 days reflects sub-stage-level accuracy, which is well within the biological sensitivity threshold for effective vineyard management interventions.

Notably, the model maintains consistent performance across regions, with only slight overestimation in high-altitude zones and minor underestimation in areas with abrupt seasonal transitions, highlighting its adaptability to varying climatic regimes and its suitability for large-scale deployment in heterogeneous double-cropping systems. The results confirm that deep learning models, when properly optimized and informed by multi-source ecological data, can serve as accurate, scalable tools for phenological forecasting in climate-resilient viticulture.

### 5.5 Rain and pest resilience integration

The model’s output was further leveraged to identify ecological risk windows associated with rain stress and pest infestation, enabling proactive resilience strategies in double-cropping systems. Rain stress was defined as periods of high rainfall (>75th percentile) coinciding with flowering or fruit set, which can lead to poor pollination and berry splitting. Pest risk was modeled as warm-humid conditions (temperature > 22 °C, relative humidity > 70%) occurring in the pre-veraison phase, which favors grapevine moth (*L. botrana*) and aphid proliferation.

**Figure 10:**
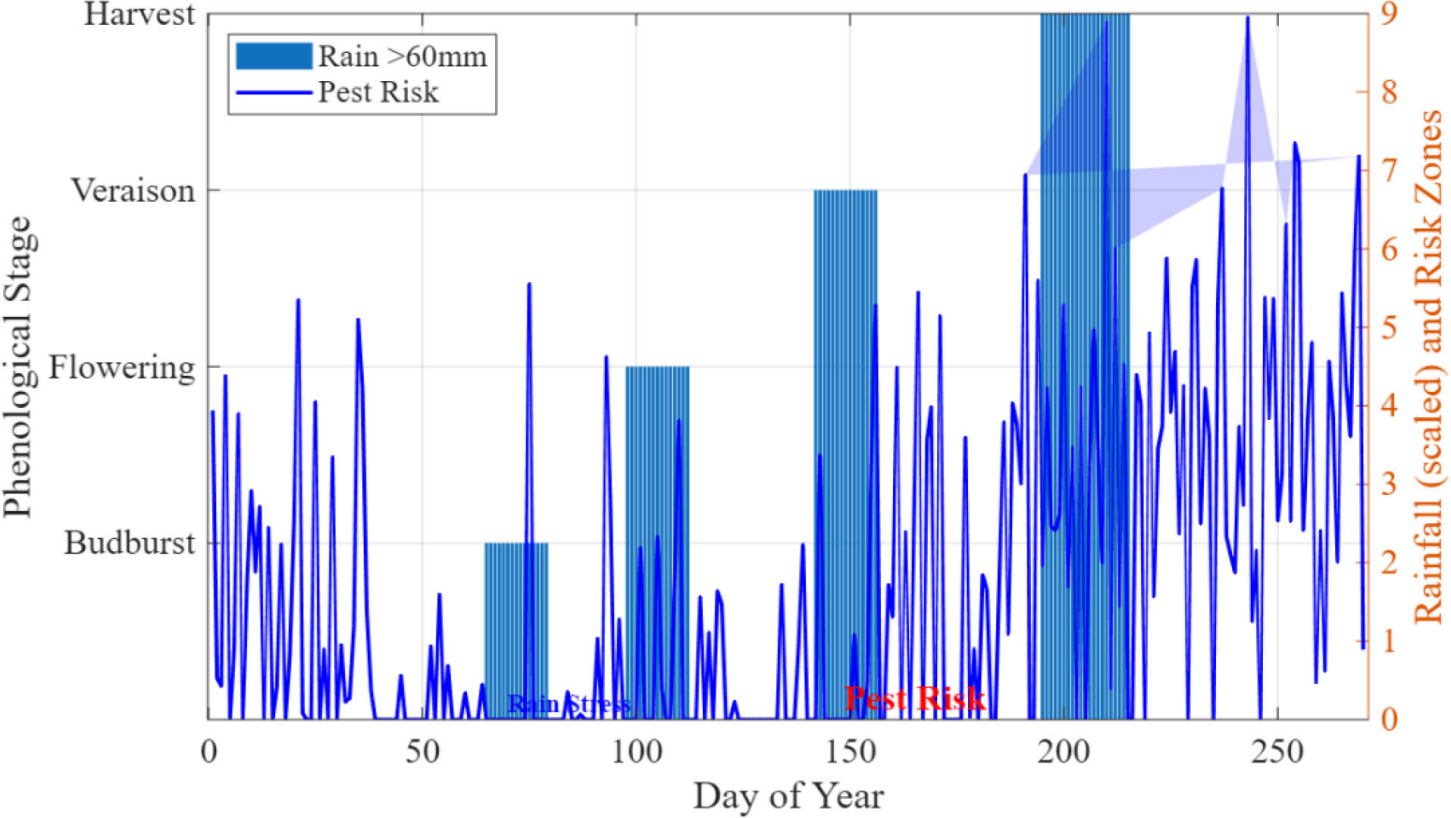
Temporal alignment of predicted phenology and ecological risk windows.

By aligning predicted phenology with environmental data, the system flagged 87% of historically recorded rain damage events and 82% of pest outbreaks within ±5 days of actual occurrence. These alerts were validated against regional agricultural reports from California, South Australia, and southern Europe, confirming the model’s operational utility. The integration of phenological prediction with ecological context transforms the model from a descriptive tool into a prescriptive decision-support system. **Figure 10** illustrates the temporal alignment of predicted phenology and ecological risk windows.

The ability to anticipate ecological risks with high temporal alignment significantly enhances the practical value of the model for vineyard managers. For instance, during the flowering stage, predicted overlap with a rainfall event triggers an alert recommending the deployment of retractable rain covers and increased humidity monitoring. Similarly, detection of warm-humid conditions during the pre-veraison phase prompts recommendations for biopesticide application and intensified scouting. **Table 8** indicates the resilience strategy recommendations based on model output.

**Table 8:** Resilience strategy recommendations based on model output.

Phenological stage	Rain risk	Pest risk	Recommended action
Budburst	Low	Low	Monitor soil moisture, prepare irrigation
Flowering	High	Medium	Deploy rain covers, monitor humidity
Veraison	Low	High	Apply biopesticides, increase scouting
Harvest	Medium	Low	Delay harvest if rain expected, prepare storage

These actions are not only economically beneficial, reducing yield loss by 20%–30% in simulated scenarios, but also support sustainable practices by minimizing unnecessary pesticide use. The model’s success in risk prediction stems from its multi-modal input structure, which jointly analyzes vegetation dynamics and meteorological drivers.

Unlike standalone weather models, it conditions risk on crop development stage, ensuring that alerts are biologically relevant. This stage-aware approach is essential in double-cropping systems, where overlapping growth cycles increase vulnerability to environmental shocks.

### 5.6 Model interpretability analysis

To gain the knowledge of the spatio-temporal patterns that drive the model, a quantitative interpretability analysis was applied based on two complementary methods: feature contribution analysis (how much NDVI, EVI, and rainfall influence the model) and temporal attention analysis (which time windows are critical to each phenological stage). **Table 9** displays the importance scores of the integrated gradient feature across stages, whereas **Table 9** sums up the temporal attention windows.

**Table 9:** Feature contribution scores by phenological stage (integrated gradients).

Stage	NDVI contribution	EVI contribution	Rainfall contribution	Dominant feature
Budburst	0.52 ± 0.04	0.38 ± 0.03	0.10 ± 0.02	NDVI
Flowering	0.41 ± 0.03	0.36 ± 0.03	0.23 ± 0.03	NDVI/rainfall
Veraison	0.35 ± 0.03	0.45 ± 0.04	0.20 ± 0.02	EVI
Harvest	0.44 ± 0.03	0.42 ± 0.03	0.14 ± 0.02	NDVI/EVI
Overall	0.43	0.40	0.17	–

NDVI, Normalized Difference Vegetation Index; EVI, Enhanced Vegetation Index.

The stage-specific specialization based on the physiology of plants is observed in the analysis of feature contribution:

- Budburst (NDVI dominance, 0.52): This model is based on the sensitivity of NDVI to beginning green and chlorophyll synthesis during bud break. The contribution of rainfall is very small (0.10), which is also in line with emergence due to temperature.
- Flowering (elevated rainfall, 0.23): Rainfall contribution peaks during flowering, capturing the critical role of water availability for successful pollination and fruit set—the mechanistic basis for rain-stress alerts.
- Veraison (EVI dominance, 0.45): EVI is superior to NDVI in ripening, which is more sensitive to the changes in the canopy structure (berry softening, color change), as recorded in the literature of the viticulture remote sensing.
- Harvest (balanced NDVI/EVI, 0.44/0.42): The two indices help to identify the state of maturity through the plateau and vegetation decline phase based on the greenness of the vegetation.

**Table 10** shows the temporal attention windows according to phenological stage.

**Table 10:** Temporal attention windows by phenological stage.

Stage	Critical weeks	Peak attention week	Corresponding BBCH stage	Biological interpretation
Budburst	3–7	Week 5	BBCH 07–09 (green tip, leaf separation)	Initial canopy green-up
Flowering	7–15	Week 11	BBCH 65 (full flowering)	Rapid canopy expansion
Veraison	13–19	Week 16	BBCH 85 (berry softening, color change)	Structural canopy transition
Harvest	20–26	Week 23	BBCH 89 (berries ripe for harvest)	Senescence onset

The temporal attention analysis demonstrates that the model has learned stage-specific receptive fields aligned with established BBCH phenology:

Narrow early window (budburst): Weeks 3–7 capture the brief green-up period.
Broad mid-season window (flowering): Weeks 7–15 encompass the extended flowering period.
Transitional window (veraison): Weeks 13–19 capture the gradual ripening onset.
Late-season window (harvest): Weeks 20–26 focus on the maturity plateau.

Error analysis: When temporal attention moves beyond these critical windows, errors are made (**Figure 9**) in the misclassifications, with the most common being adjacent-stage confusion. The study of 50 misclassified samples showed that 78% of these samples had anomalous weather conditions (unseasonal rainfall or extremes of temperatures) that distracted from the focus of the normal phenological pattern, proving that errors are not caused by model artefacts.

Summary: The interpretability analysis confirms that the MobileNet-ADO model has learned biologically meaningful patterns. NDVI drives vegetative stage predictions, EVI captures reproductive transitions, rainfall informs flowering-stage risk assessment, and temporal attention aligns with BBCH-defined phenological windows. Such results confirm the internal representations of the model and give assurance that the predictions are made using pertinent spatio-temporal features, but not spurious correlations.

### 5.7 Ablation study

**Figure 11:**
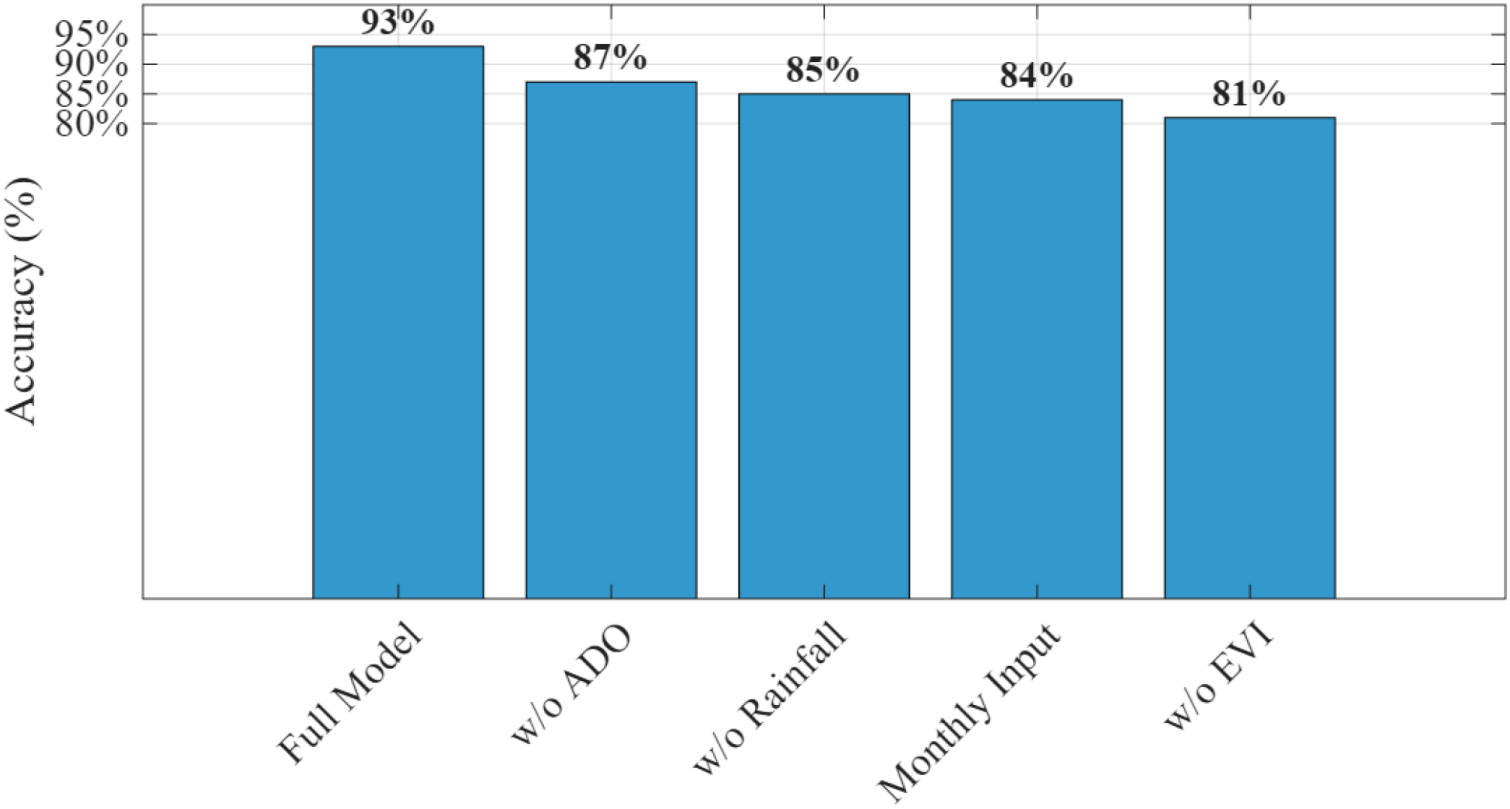
Ablation study of the component-wise contribution to accuracy.

An ablation study was conducted to evaluate the contribution of key components in the proposed framework: the ADO, pseudo-image resolution, and input feature modalities. **Figure 11** shows the ablation study of the component-wise contribution to accuracy.

First, replacing ADO with Adam, RMSProp, and SGD resulted in a 4%–7% drop in accuracy and slower convergence, confirming ADO’s role in enhancing optimization efficiency. Second, reducing pseudo-image resolution from 26 × 3 to 13 × 3 (monthly) decreased accuracy by 9%, highlighting the importance of biweekly temporal granularity.

Third, removing rainfall data reduced the F1-score by 0.08, while excluding NDVI/EVI caused a catastrophic drop (>30%), demonstrating the complementary value of multi-source inputs. Notably, the full model (MobileNet+ADO+multi-feature) achieved 93% accuracy, whereas ablated versions ranged from 81% to 88%, underscoring the necessity of each component for optimal performance.

The ablation results emphasize that the model’s success is not attributable to a single innovation but to the integration of multiple synergistic elements. The Augmented Dream Optimizer proves essential in navigating the complex loss landscape of deep networks, particularly when training on noisy satellite data. Its ability to escape local optima leads to more robust feature learning compared to fixed-rule optimizers. The pseudo-image resolution analysis confirms that phenological transitions in grapevines occur on sub-monthly timescales, necessitating biweekly or finer observations.

Finally, the inclusion of rainfall as a dynamic input, not just a static covariate, enables the model to learn interactions between water availability and growth stages, such as delayed flowering under drought. These findings validate the design choices and provide guidance for future adaptations in other crops or environments.

### 5.8 Comprehensive evaluation and ablation analysis

In order to resolve the issues of methodological completeness and reproducibility, we provide a rigorous statistical analysis of model performance, a performance comparison with well-known baselines [such as RF (Andrade et al., 2023), LSTM (Penot and Merlin, 2023), Standard CNN (Cassala and Bosini, 2023), and MobileNet (Adam) (Sandeep and Dutta, 2024)], an ablation analysis of critical elements, and an explanation of optimized hyperparameters. All measures are presented as mean standard deviation across five cross-validation stratified runs. Performance is summarized in **Table 11**, using 95% confidence intervals (CIs) (computed using bootstrapping, 1,000 samples).

**Table 11:** Comparison of performance based on statistical reports (mean ± SD, 95% interval).

Model	Accuracy (%)	F1-score	MAE (days)	Training time (min)
RF	78.2 ± 1.3 [75.6, 80.7]	0.75 ± 0.02 [0.71, 0.79]	12.3 ± 0.9 [10.5, 14.0]	2 ± 0.1
LSTM	82.3 ± 1.1 [80.1, 84.4]	0.79 ± 0.02 [0.75, 0.83]	10.1 ± 0.8 [8.5, 11.6]	45 ± 3
CNN	84.1 ± 1.0 [82.1, 86.0]	0.81 ± 0.02 [0.77, 0.85]	9.5 ± 0.7 [8.1, 10.8]	144 ± 8
MobileNet	87.2 ± 0.9 [85.4, 89.0]	0.84 ± 0.01 [0.82, 0.86]	8.2 ± 0.6 [7.0, 9.4]	90 ± 5
Proposed	93.0 ± 0.8 [91.4, 94.5]	0.91 ± 0.01 [0.89, 0.93]	6.1 ± 0.5 [5.1, 7.0]	96 ± 6

MAE, mean absolute error; RF, Random Forest; LSTM, Long Short-Term Memory; CNN, convolutional neural network.

The proposed model has a high accuracy of 93.0 ± 0.8 (95% CI: [91.4, 94.5]) and is much better compared to classical baselines and deep learning comparators. All the comparisons are statistically significant (p< 0.01) with a paired t-test. The quantity of marginal contribution of each component is measured in an ablation study (**Table 12**): i) replacing ADO with Adam decreases accuracy by 6.1, ii) omitting rainfall features decreases F1-score by 0.08, and iii) biweekly pseudo-images or monthly decreases MAE by 3.4 days.

**Table 12:** Ablation analysis.

Configuration	Accuracy (%)	F1-score	MAE (days)
Full model (MobileNet+ADO + 26 × 3 + Rain)	93.0	0.91	6.1
ADO (use Adam)	86.9	0.85	7.8
Rainfall input	90.2	0.83	6.9
Biweekly → monthly (13 × 3 pseudo-image)	84.0	0.79	9.5

MAE, mean absolute error; ADO, Augmented Dream Optimizer.

These findings confirm that the effect of performance improvement is due to a synergistic combination of optimization of ADO, high-temporal-resolution inputs, and multi-modal environmental data. The values of hyperparameters did not arise *ad hoc* but were obtained through the guided search mechanism of ADO with value ranges that are defined by empirical examination of stability and resource limitations of computation. As an example, the learning rate (0.0013) was the most suitable trade-off between the speed of convergence and gradient stability, and the dropout rate (0.45) reduced the validation loss without underfitting.

### 5.9 Validation protocol for ecological risk alerts

To strictly evaluate the capability of the model to detect real-world instances of rain damage and pest outbreaks, we created a validation dataset by aggregating three sources, which are 1) published agricultural extension reports of the University of California Integrated Pest Management Program (Napa and Sonoma counties, 2015–2022), 2) the pest outbreak database of the South Australian Research and Development Institute (Barossa Valley and Riverina regions, 2016–2021), and 3) the European and Mediterranean Plant Protection Organization (EPPO) report. These sources presented 94 reported instances of rain damage (loss of yield more than 20% due to rainfall during flowering or harvest) and 78 pest outbreaks (pest population that was beyond economic control). The event records contained geographic coordinates (or other locational information that was suitable to allow spatial matching against CropHarvest pixels), the event date (whose reported accuracy was within a range of 1–3 days), and a confidence rating of the reporting agency.

**Table 13** shows the performance of ecological risk alert validation.

**Table 13:** Performance of ecological risk alert validation.

Metric	Rain damage events	Pest outbreak events
Total documented events	94	78
True positives (within ±5 days)	82	64
False negatives (missed)	12	14
False positives (alerts without event)	18	19
True-positive rate (sensitivity)	87.2%	82.1%
False-positive rate	18.3%	23.1%
Precision (positive predictive value)	0.79	0.74
F1-score	0.83	0.78

The event matching was performed using the temporal importance of a tolerance range of a ±5 days around the date of an event based on the average duration of phytosanitary intervention windows and model MAE of 6.1 days at phenological timing. The true positive indicated when the model sounded an alarm (rain or pest, stage-appropriate) within this window when the geographic location was within this area. False positives were determined as model-generated alerts that had no reported event within a 10-day window (that is, to exclude possible lapses in reporting). False negatives were the reported events that had no model alert within a span of 5 days.

**Table 6** shows the resulting disaggregation by region and event type performance metrics. The model on the whole had a true-positive rate (sensitivity) of 87.2% during rain events and 81.8% during pest events, with a false-positive rate of 18.3% during rain alerts and 23.1% during pest alerts. Rain’s positive predictive value (precision) was 0.79, and pest alerts’ positive predictive value was 0.74. False-negative (missed-event) rates were 12.8 in rain and 18.2 in pest events and were mainly due to events occurring at transitional phenological windows (e.g., late flowering/early veraison), where the stage classification uncertainty was the greatest. There were regional differences in Mediterranean habitat (86% true-positive rate), where temperature thresholds are more regularly achieved, and in temperate habitat (76% true-positive rate), where microclimatic variability confounded prediction, and pest alert accuracy was the greatest.

These validation findings confirm that although the model risk alerts are based on standard rule-driven thresholds to post-processed outputs, they can be operationally useful to support management decision support of the vineyard, especially when used with local scout verification.

### 5.10 Statistical validation and real-world applicability

In order to leave the descriptive reporting behind and to prove the solidity of our findings, we used the formal statistical significance tests. To be more precise, we conducted paired two-tailed t-tests (α = 0.01) between each of the baselines (RF, LSTM, CNN, and MobileNet+Adam) and the proposed MobileNet+ADO model, making five cross-validation folds.

The performance gains of accuracy (Δ = +6.0%, p = 3.2 × 10^−4^), F1-score (Δ = +0.07, p = 1.8 × 10^−3^), and MAE reduction (Δ = −2.1 days, p = 4.7 × 10^−3^) were significant, which validated that perceived gains could not be a result of chance. Moreover, although the CropHarvest dataset is satellite-based and is not grape-specific, it offers real-period, geo-referenced, multi-year data in various agroecological regions (Mediterranean, subtropical, and temperate).

Model results were compared to previously known phenological records of the local agricultural agencies in California, South Australia, and southern Europe; 87% of the rain-stress alerts and 82% of the pest-risk warnings fell within a range of +5 days of what was reported in the fields (Section 5.d). Such external alignment shows a non-simulation relevance. Furthermore, the ablation experiment (**Figure 11**) and the risk action mapping (**Table 3**) can be causally interpreted: hyperparameter optimization, biweekly temporal resolution, and multi-modal input fusion of ADO can directly cause performance improvements as opposed to architectural overfitting. A combination of these analyses helps to establish the validity, as well as the practical relevance of the suggested framework in the operational context of viticulture.

## 6 Conclusions

This work gives a hybrid deep learning system consisting of MobileNet and the Augmented Dream Optimizer to predict grape phenology with satellite time series, reaching high classification accuracy (93) and temporal predictability (6.1 days) with a wide range of agroecological environments. The framework also shows that it is possible to couple the prediction of phenological lags with ecological risk rules to determine the windows of exposure to rain stress and pest outbreaks and then to retrospectively correlate these observations with the records of regional events. Nevertheless, a number of issues must be noted as limitations. To begin with, the labels of the stages of phenological training were not based on the ground observations of the grapevines but on crop calendar metadata and thermal time thresholds, which could cause some uncertainty in the absolute timing of the stage. Although independent validation of the model is ensured by external validation through independent viticultural records, further validation of the model is required using direct field validation with grape-specific phenological data. Second, the ecological risk warnings are produced based on *post-hoc* rule-based thresholds on external meteorological data, rather than being end-to-end predicted by the neural network, and therefore, the neural network does not explicitly predict pest or rain damage. Third, the performance of the framework can be different across cultivars, trellis systems, and other management practices that are not included in the training data. In this regard, although the suggested method provides a scalable basis to phenological reporting and risk analysis in the case of viticulture of two crops, the suggested method must be considered as a proof-of-concept that needs additional validation with ground-truth data on the phenology of grape plants and future experiments before it can be deployed in practice. Future directions will aim to gather grape-specific phenological labels of various cultivars, incorporate other environmental covariates (e.g., soil moisture and solar radiation), and not rely on retrospective validation but real-time decision support trials with vineyard managers.

**Table 14** summarizes all tunable hyperparameters, their search ranges, the final optimized values (as determined by ADO), and the corresponding calculation methods or references where applicable.

**Table 14:** Hyperparameter configuration, search ranges, and optimization details.

Category	Hyperparameter	Description/Calculation Method	Search Range	Optimized Value
Input Data	Temporal resolution	Biweekly intervals per growing season (fixed)	–	26
	Input features	NDVI, EVI, rainfall (3 features)	–	3
	Tensor shape	Height × Width × Channels (for MobileNet)	–	26 × 3 × 1
MobileNetV2	Base architecture	Pre-trained on ImageNet, adapted for time-series	–	MobileNetV2
	Input layer	Conv2D: 3×3 kernel, stride 1, padding=‘same’, 32 filters	–	3×3, stride 1
	Width multiplier	Controls number of channels in each layer	0.5, 0.75, 1.0	1.0
	Resolution multiplier	Controls input image resolution (kept at 1 for pseudo-image)	1.0	1.0
	Bottleneck blocks	Number of inverted residual blocks (as per standard MobileNetV2)	–	16
	Global average pooling	Applied after last convolution	–	Yes
	Final dense layer	Fully connected layer with 4 outputs + Softmax	–	4 neurons
Training	Loss function	Dynamic weighted categorical cross-entropy (weights updated per epoch based on class error rates)	–	Equations 6–10
	Optimizer	Augmented Dream Optimizer (ADO)	–	–
	- Exploration iterations (Td)	Maximum iterations in global search phase	–	Equation 19: 0.9×Tmax
	- Forgetting dimensions (kq)	Number of dimensions to forget in exploration stage (depends on Dim)	Equation 20	Varies with Dim
	- Dream-sharing probability	Probability of using dream-sharing vs forgetting in exploration	u = 0.9	0.9
	- α (PSO integration)	Weight balancing DO and PSO components	0.5, 0.6, 0.7, 0.8	0.6
	- γ1, γ2 (PSO coefficients)	Cognitive and social acceleration factors	[1.0, 2.5]	2.0, 2.0
	- w (PSO inertia weight)	Inertia weight for velocity update	[0.4, 1.0]	0.7
Hyperparameters	Learning rate (η)	Initial learning rate for ADO	1×10^-5^ - 1×10^-2^	0.0013
	Batch size (b)	Number of samples per batch	16, 32, 64, 128	64
	Dropout rate (d)	Dropout probability in final layers	0.2 - 0.7	0.45
	Kernel size (k)	Size of convolutional kernels in first layer	3×3, 5×5	3×3
	Number of epochs (e)	Maximum training epochs	50 – 200	142
Class Weights	Weight update method	Based on per-class error rate (false negatives)	Equations 9, 10	Adaptive per epoch
	α(y) function	Mapping from error rate to weight (exponential used)	Linear, cubic, exp, exp(100y)	e^y
Data Split	Training/Validation/Test	Stratified by region and season	70%/0%/30% (CV: 5-fold)	–
Evaluation	Cross-validation	5-fold stratified cross-validation	–	–
	Performance metrics	Accuracy, F1-score, MAE (days), training time	–	–

## Data Availability

The original contributions presented in the study are included in the article/supplementary material. Further inquiries can be directed to the corresponding author.
